# Plant species identity drives soil microbial community structures that persist under a following crop

**DOI:** 10.1002/ece3.6560

**Published:** 2020-07-23

**Authors:** Aaron Fox, Andreas Lüscher, Franco Widmer

**Affiliations:** ^1^ Forage Production and Grassland Systems Agroscope Zürich Switzerland; ^2^ Molecular Ecology Agroscope Zürich Switzerland

**Keywords:** AMF/Glomeromycota, legacy effect, metabarcoding, plant species identity, plant–soil interaction

## Abstract

Compared to monocultures, multi‐species swards have demonstrated numerous positive diversity effects on aboveground plant performance, such as yield, N concentration, and even legacy effects on a following crop. Whether such diversity effects are seen in the soil microbiome is currently unclear. In a field experiment, we analyzed the effect that three plant species (a grass, forb, and legume), and mixtures of these, had on soil fungal and bacterial community structures, as well as their associated legacy effects under a following crop, the grass *Lolium multiflorum*. We utilized six sward types, three monocultures (*Lolium perenne*, *Cichorium intybus* and *Trifolium pratense*), two bi‐species mixtures, and a mixture of the three species. Soil samples were taken from these swards in March (at the end of a three year conditioning phase) and in June, August, and September after *L. multiflorum* was established, that is, the legacy samplings. When present, the differing monocultures had a significant effect on various aspects of the fungal community: structure, OTU richness, the relative abundance of the phylum Glomeromycota, and indicator OTUs. The effect on bacterial community structure was not as strong. In the multi‐species swards, a blending of individual plant species monoculture effects (identity effect) was seen in (a) fungal and bacterial community structure and (b) fungal OTU richness and the relative abundance of the Glomeromycota. This would indicate that plant species identity, rather than diversity effects (i.e., the interactions among the plant species), was the stronger determinant. During the legacy samplings, structural patterns in the fungal and bacterial communities associated with the previous swards were retained, but the effect faded with time. These results highlight that plant species identity can be a strong driver of soil microbial community structures. They also suggest that their legacy effect on the soil microbiome may play a crucial role in following crop performance.

## Graphical abstract

We investigated the effect of differing plant functional types, that is, grass, forb, and legume as well as various combinations of these, and their associated legacy effects on microbial community structure using amplicon‐based metagenomics.

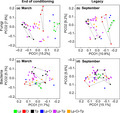

## 1 INTRODUCTION

**Figure 1:**
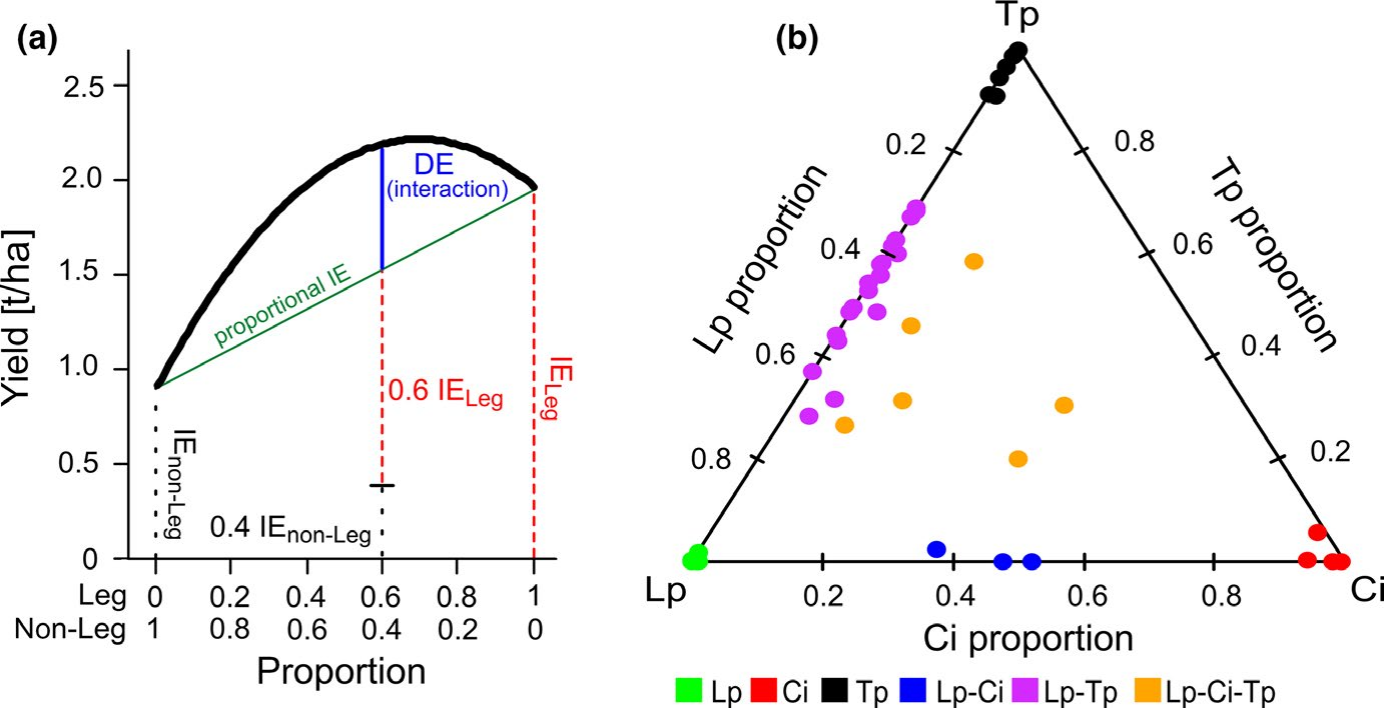
(a) A conceptualization of the expected response of the yield of bi‐component mixtures based exclusively on the identity effects of the two components (IE, green line) and, if in addition to that, the two components show (positive) interactions, the so called diversity effect (DE, blue line). For the purposes of this example, the biomass yield of the following crop (*Lolium multiflorum*) at the legacy harvest in June is displayed (Fox et al., 2020, i.e., the same experimental system and sampling times as discussed in this study), at varying proportions of the two components legumes (Leg) and nonlegumes (non‐Leg) of the previous sward. For further conceptual details on identity and diversity effects in mixtures and their analyses based on a simplex design, consult Kirwan et al., 2009. (b) Experimental design, following a simplex design, displaying the realized proportion of the three plant species in each of the 45 experimental plots used for the present study at the end of the three‐year conditioning phase in March. Abbreviations for sward type names are as follows; *Lolium perenne* monoculture: Lp, *Cichorium intybus* monoculture: Ci, *Trifolium pratense* monoculture: Tp, *Lolium*–*Cichorium* bi‐species sward: Lp–Ci, *Lolium*–*Trifolium* bi‐species sward: Lp–Tp, and *Lolium*–*Cichorium*–*Trifolium* tri‐species sward: Lp–Ci–Tp

The adoption of multi‐species swards with few, but complementary plant species (particularly grass‐legume mixtures), has been advocated as a sustainable alternative in intensively managed grasslands (Lüscher, Mueller‐Harvey, Soussana, Rees, & Peyraud, 2014). They have demonstrated numerous agronomic and environmental advantages in comparison with conventional, highly fertilized, grass monocultures (i.e., Connolly et al., 2018; Finn et al., 2013; Nyfeler, Huguenin‐Elie, Suter, Frossard, & Lüscher, 2011; Suter et al., 2015). This is due to complementarity of resource acquisition strategies among different plant functional traits in multi‐species swards (Hooper et al., 2005; Loreau et al., 2001), such as seasonality of growth (Husse, Lüscher, Buchmann, Hoekstra, & Huguenin‐Elie, 2017), rooting depth (Hoekstra, Suter, Finn, Husse, & Lüscher, 2015), and N_2_‐fixing capability (Nyfeler et al., 2011; Suter et al., 2015). Importantly, these positive effects of plant species diversity (diversity effects) derive from (positive) interactions among the plant species and, thus, are more than just the proportional contribution of the measured performances of each species as a monoculture (their identity effect), as conceptualized in Figure 1a. The role the soil microbiome plays in diverse plant swards, however, is coming increasingly into focus (Kardol, De Deyn, Laliberté, Mariotte, & Hawkes, 2013; Schnitzer et al., 2011). Soil microorganisms are instrumental in a number of fundamental ecosystem processes such as organic matter decomposition and nutrient cycling, as well as plant productivity (Schnitzer et al., 2011; Wagg, Bender, Widmer, & van der Heijden, 2014).

How different plant species of different functional types, that is, grasses, forbs and legumes, and their combinations influence their associated microbial communities is receiving increased attention (e.g., Ladygina & Hedlund, 2010; Zhou, Zhu, Fu, & Yao, 2017). Different plant species have been shown to induce distinct microbial community structures within a given soil type (Leff et al., 2018). The contrasting physiologies and traits of different plant species, that is, root volume and length, root respiration, and particularly chemical composition and deposition patterns of root exudates, are likely key drivers of such differences. The plant species *Lolium perenne* (Lp)*, Cichorium intybus* (Ci), *and Trifolium pratense* (Tp) differ both functionally and structurally. They are functionally distinct on account of the ability of Tp to symbiotically fix N_2_ (Nyfeler et al., 2011), while Lp and Ci cannot. Overtime, the N‐rich rhizodeposits and residues of Tp may alter soil organic matter (SOM) chemistry and lower its C/N ratio (Hammelehle, Oberson, Lüscher, Mäder, & Mayer, 2018). Additionally, while root exudate components from grasses and forbs have been found to be largely the same, some metabolites have been shown to be unique to either functional group (Herz et al., 2018). Such differences may influence microorganisms which are involved in the soil N cycle, such as the Nitrospirae (Lücker et al., 2010; Zhou et al., 2017) and those, which are influenced by changes in SOM lability such as the Bacteroidetes (Fierer, Bradford, & Jackson, 2007) and Planctomycetes (Buckley, Huangyutitham, Nelson, Rumberger, & Thies, 2006). The rooting architectures among the three plant species also differ. The root structure of Ci and Tp are characterized by a deep tap root, while Lp has a shallow, filamentous root system (Black, Laidlaw, Moot, & O'Kiely, 2009; Brown, Moot, & Pollock, 2003; Hoekstra et al., 2015). Additionally, the root biomass of Lp and Ci is greater than that of Tp (Hofer, Suter, Buchmann, & Lüscher, 2017). Such differing root systems may lead to differing C‐deposition patterns in the soil matrix, as well as influence the root colonization rate of arbuscular mycorrhizal fungi (Ryan, Small, & Ash, 2000). Physiological differences between plant species, particularly in litter quality, has been suggested as the reason why plant functional group identity (particularly legumes) was the stronger driver of soil microbial community composition, compared to plant species richness, in the Jena biodiversity experiment (Dassen et al., 2017). Nevertheless, strong diversity effects deriving from plant species interactions, in intensively managed fertile grasslands, on aboveground plant performance, such as biomass yield (Finn et al., 2013; Nyfeler et al., 2009), N uptake from symbiotic and soil sources (Nyfeler et al., 2009; Suter et al., 2015) and soil‐transferred legacy effects (Fox, Suter, Widmer, & Lüscher, 2020), would suggest that besides plant species identity effects on soil microorganisms, significant plant diversity effects could also be expected (Figure 1a). A greater understanding, however, as to how and to what extent plant identity and diversity (determined by species richness and evenness) influence the structure of the soil microbiome is still required, particularly under field conditions.

As sown grassland is often utilized as a ley in crop rotations, any persistent effects they impart on the soil microbiome, and thus on a following crop, may have important agro‐ecological implications. Plant residues and root exudates, which are deposited and shed into the soil matrix, can persist and have lasting implications in crop rotations. Beneficial legacy effects, associated with a preceding legume crop (such as the annual pea plant *Pisum sativum*), have been documented on following cereal crop performance in arable systems (Evans et al., 1991; Stevenson & van Kessel, 1996). Positive legume‐associated legacy effects have also recently been demonstrated for a following grass crop (*Lolium multiflorum*) after a three year ley (Fox et al., 2020). As these preceding crops were removed from the system, these legacy effects must be soil transferred. Legacy‐associated plant residues may lead to the persistence of microbial groups which are best suited to utilize those (Allison et al., 2013). Previous research has also illustrated the legacy effect that differing species of grassland plants have various trophic levels of soil biology, that is, fungi, nematodes, mites, and earthworms, in a rotational agronomic system (Crotty et al., 2016; Detheridge et al., 2016). What is currently not clear, however, is the extent and the persistence of the influence imparted by different sward types (i.e., monocultures of different species and their mixtures) on soil bacterial and fungal community structure, and whether both constituents of the microbiome respond in a similar manner. Components of the soil microbiome have been shown to play important functional roles in the legacy effects on a following crop (Hannula, Ma, Pérez‐Jaramillo, Pineda, & Bezemer, 2020).

This study had two principal objectives. Firstly, to determine the effect of both plant species identity and diversity (varied by richness and relative abundance of the different plant species) on soil microbial community structures. Secondly, to analyze whether such effects persisted after the swards were removed and a new following crop was established.

Specifically, we tested the following hypotheses:

1. Different plant species identities differently influence the structure of soil fungal and bacterial communities.
2. Plant species identity is a stronger determinant of soil fungal and bacterial richness than plant diversity.
3. Structural patterns of soil bacterial and fungal communities associated with previous sward types persist under a new crop, for a certain time period.

## 2 MATERIALS AND METHODS

### 2.1 Three year soil conditioning phase

For this present field experiment, we made use of plots from previous plant mixture experiments (consult Hofer et al., 2017; Hofer et al., 2016) that conditioned the soil, a cambisol (top soil 42% silt, 26% clay, pH = 7.1). The field was located at the Agroscope Station‐Reckenholz in Zurich (47°26′N, 8°32′E, 491 m a.s.l, mean annual temperature: 9.4°C, mean annual precipitation 1,031 mm). These swards were maintained over a period of three years (2012–2015), a typical duration of a ley in a crop rotation, which provided the conditioning required for the present study. Briefly, this conditioning phase used four perennial model plant species, which represent key species for intensively managed temperate grasslands worldwide and are characterized by their factorial combination of functional traits related to rooting depth and symbiotic N_2_‐fixation, that is, the shallow rooting grass *Lolium perenne* L. (Lp), the deep rooting forb *Cichorium intybus* L. (Ci), the shallow rooting legume *Trifolium repens* L. (white clover), and the deep rooting legume *Trifolium pratense* L. (red clover, Tp). For additional details, see Hofer et al. (2016), Hofer et al. (2017). This experiment varied plant species richness, as well as the relative abundance of each species within the sward over a wide range according to a simplex design (Cornell, 2002), the strength of which is to separate species identity effects and diversity effects in multi‐species swards (Figure 1a; Connolly et al., 2013; Kirwan et al., 2009). Plots (3 × 5 m) were sown as monocultures (100% of each species; 10 replicates each = 40 plots), bi‐species mixtures (50% each of two species; 8 replicates each = 48 plots), an equi‐proportional mixture (25% of each of the four species; 10 replicates each = 10 plots), and a dominant mixture (79% of the dominant species, 7% each of the other three species; 4 replicates each = 16 plots), resulting in a total of 114 plots. At the end of the three year conditioning phase, species composition of the swards had changed substantially compared to the sowing rates as was also observed in other studies (Brophy et al., 2017). This was especially the case for *Trifolium repens* L., which had drastically declined in the swards after being outcompeted by the other species.

### 2.2 Experimental setup and soil sampling

From the 114 originally sown swards as described above, 45 were selected to examine the persistent effects of plant species identity and diversity on the structure of soil microbial communities. Due to its low abundance, *Trifolium repens* L., was no longer considered in this study (highest abundance 3.5%). As shown in Figure 1b, there were three different monoculture swards (defined as those, which were sown as monocultures and still contained > 90% biomass proportion of the sown plant species, including unsown species); Lp (*n* = 6), Ci (*n* = 4), and Tp (*n* = 7). There were also multi‐species swards. These were two bi‐species swards; Lp–Ci (*n* = 3) and Lp–Tp (*n* = 19), where the relative abundance of a plant species varied widely from 25% to 70%), and a tri‐species containing sward, containing the three plant species (in which a species was >10% abundant, Lp–Ci–Tp, *n* = 6). A Ci–Tp sward was not included due to the absence of plots with the adequate plant composition after the conditioning phase. Henceforth, these six swards shall be collectively referred to as "sward type."

On the 16 March 2015, when the swards were still present (hereafter referred to as the end of the conditioning phase), eight soil samples were taken from each plot using a 2.5 cm auger to a depth of 20 cm. The cores from each plot were combined and then sieved (2 mm) with each sample being thoroughly homogenized. Approximately 0.5 g of each sample was then added to a 2‐ml Eppendorf tube which contained 0.5 g of glass beads (ø 0.10–0.11 mm). After adding 1.2 ml extraction buffer (0.2 M Na_3_PO_4_ of pH 8, 0.1 M NaCl, 50 mM EDTA, 0.2% CTAB), the tubes were vortexed and stored at −20°C.

On April 21, these swards were completely removed using a glyphosate herbicide (Roundup**^®^**, Monsanto, MI), and thereby terminating the conditioning phase of the experiment. All plots were re‐seeded after preparation with a rotary harrow with a monoculture of *Lolium multiflorum* L., a grass. This following sward was allowed to become established and soil samples were taken in three subsequent legacy samplings on June 10, August 25 and September 29 (referred to as legacy samplings subsequently). Soils from these samplings were processed as previously described. A schematic over‐view of the experiment can be found in Figure S1. The aboveground biomass yield was harvested as part of the study outlined in Fox et al. (2020). For the purposes of the present study, the phosphorus (P) content of *L. multiflorum* from the June legacy harvest was determined via ICP‐OES.

### 2.3 DNA extraction and metabarcoding

DNA was extracted from each soil sample in three repeated extractions according to the procedure outlined previously in Bürgmann, Pesaro, Widmer, and Zeyer (2001), alongside the modifications described by Hartmann, Frey, Kölliker, and Widmer (2005). DNA extracts were then quantified using the PicoGreen dsDNA quantification assay (Invitrogen) using a Cary Eclipse fluorescence spectrophotometer (Varion Inc.). Extracts were then diluted with sterile ddH_2_O to a concentration of 5 ng/µl. The fungal internal transcribed spacer region (ITS2) of the rRNA operon was amplified by PCR using primers ITS3 (5′ CAH CGA TGA AGA ACG YRG 3′) and ITS4 (5′ TCC TSC GCT TAT TGA TAT GC 3′, Tedersoo et al., 2014), while the V3‐V4 region of the bacterial 16S rRNA gene was amplified using the primer pair 341F (5′ CCT AYG GGD BGC WSC AG 3′) and 806R (5′ GGA CTA CNV GGG THT CTA AT 3′, Frey et al., 2016). The 5′ end of the forward primer was tagged with a CS1 adapter, while the reverse primer was tagged with CS2, which allowed multiplexing with the Fluidigm Access Array System (Fluidigm). PCR amplification conditions were as previously described (Mayerhofer et al., 2017), with the modification that reaction volumes of 20 μl and 35 amplification cycles were used. Each reaction was repeated four times, then pooled and sent to the Génome Québec Innovation Center for PE‐300 sequencing using the Illumina Miseq v3 platform (Illumina Inc.).

### 2.4 Sequence processing, taxonomic classification, and alpha diversity estimation

Sequences were processed using a customized pipeline in UPARSE implemented in USEARCH version 9 (Edgar, 2010) with the specific details described previously (Frey et al., 2016; Mayerhofer et al., 2017). Taxonomic classification of operational taxonomic unit (OTU) centroids (which were clustered at 97% identity) was performed using the Bayesian classifier implemented in MOTHUR version 1.36.1 (Schloss et al., 2009), with the SILVA ribosomal RNA gene database (version 132, Quast et al., 2013) used as a reference for prokaryotic sequences, and a custom‐made database extracted from the NCBI GenBank (Benson et al., 2014) for eukaryotic sequences. Nonfungal sequences were discarded from the eukaryotic dataset, and retained fungal OTUs (fOTUs) were assigned using the UNITE reference database (version 7.2, Abarenkov et al., 2010). Lastly, prokaryotic OTUs assigned to chloroplasts, mitochondria, and archaea were removed to retain only bacterial OTUs (bOTUs) for analyses. Fungal and bacterial OTU richness and the inverse Simpson index (iS) were calculated using the "summary.single" command in the MOTHUR software. When calculating these measures, differences in sequencing depth were achieved by an iterative (10,000x) subsampling to the lowest sequence number per sample in the dataset (17,542 sequences for fungi and 18,655 sequences for bacteria, Figure S2). Simpson's evenness of both the fungal and bacterial community was highly correlated to the corresponding iS measurement and therefore was not subject to further analysis.

### 2.5 Data analysis

All statistics were performed in R (R Development Core Team, 2019). To determine the effect of sward type (which had five degrees of freedom), both the fungal and bacterial OTU abundance tables were square‐root transformed, converted to relative abundance and a Bray–Curtis dissimilarity matrix was constructed (Hartmann et al., 2012). Significance of difference in bacterial and fungal community structures was assessed with PERMANOVA using the "adonis" function within the "vegan" package (Oksanen et al., 2019). Pairwise differences of communities were determined using the "pairwise.perm.MANOVA" function in the "RVAideMemoire" package (Hervé, 2019) with Benjamini–Hochberg *p*‐value correction. The community centroids and the homogeneity among the communities in the different sward types were calculated using the "betadispr" function in the "vegan" package (Anderson, Ellingsen, & McArdle, 2006). Differences in both fungal and bacterial community structures between the different sward types at each sampling time were displayed using principal co‐ordinate analysis (PCO) plots. Plant species diversity (based on inverse Simpson, iS) was calculated via the "diversity" function in the "vegan" package, with a linear regression analysis used to determine its relationship to both fungal and bacterial OTU richness. Associations of OTUs to each of the monocultures was determined by correlation based indicator species analysis ("multiplatt" function) with 9,999 permutations in the "indicspecies" package (Cáceres & Legendre, 2009). Indicator OTUs were defined as those with an IndVal ≥ 0.8 and a *p*‐value ≤.05.

The main effect of sward type on fungal or bacterial OTU richness, that is, inverse Simpson (iS) and the relative abundance (%) of different phyla, was determined by analysis of variance (ANOVA). Differences of sward types to Lp were tested using post hoc contrasts derived from the ANOVA model (*t* tests, without applying multiple comparisons). As Lp was the most conventional sward from an agronomic standpoint, we selected it as a reference to see how adopting differing swards influenced soil microbial community structure. Moreover, because the response of fOTU richness, the relative abundance of the fungal phylum Glomeromycota in March, and P yield in June, were related to Tp abundance, the effect of Tp proportion and its associated legacy effect on these responses was analyzed via linear regression (for the end of the conditioning phase) or linear mixed effects regression (for the legacy samplings, as this was a repeated measure, Pinheiro & Bates, 2000). Both types of models included a quadratic legume proportion term to allow for flexibility in the response curves (Fox et al., 2020), and the marginal and conditional *R* ^2^ values for the linear mixed models were calculated as described in Nakagawa and Schielzeth (2013). The range of realized Tp proportions for which any of the response variables were significantly different from the value at 0% Tp proportion was calculated using the Johnson–Neyman technique (Potthoff, 2006).

## 3 RESULTS

### 3.1 Persistent differences in fungal and bacterial communities associated with sward types

**Figure 2:**
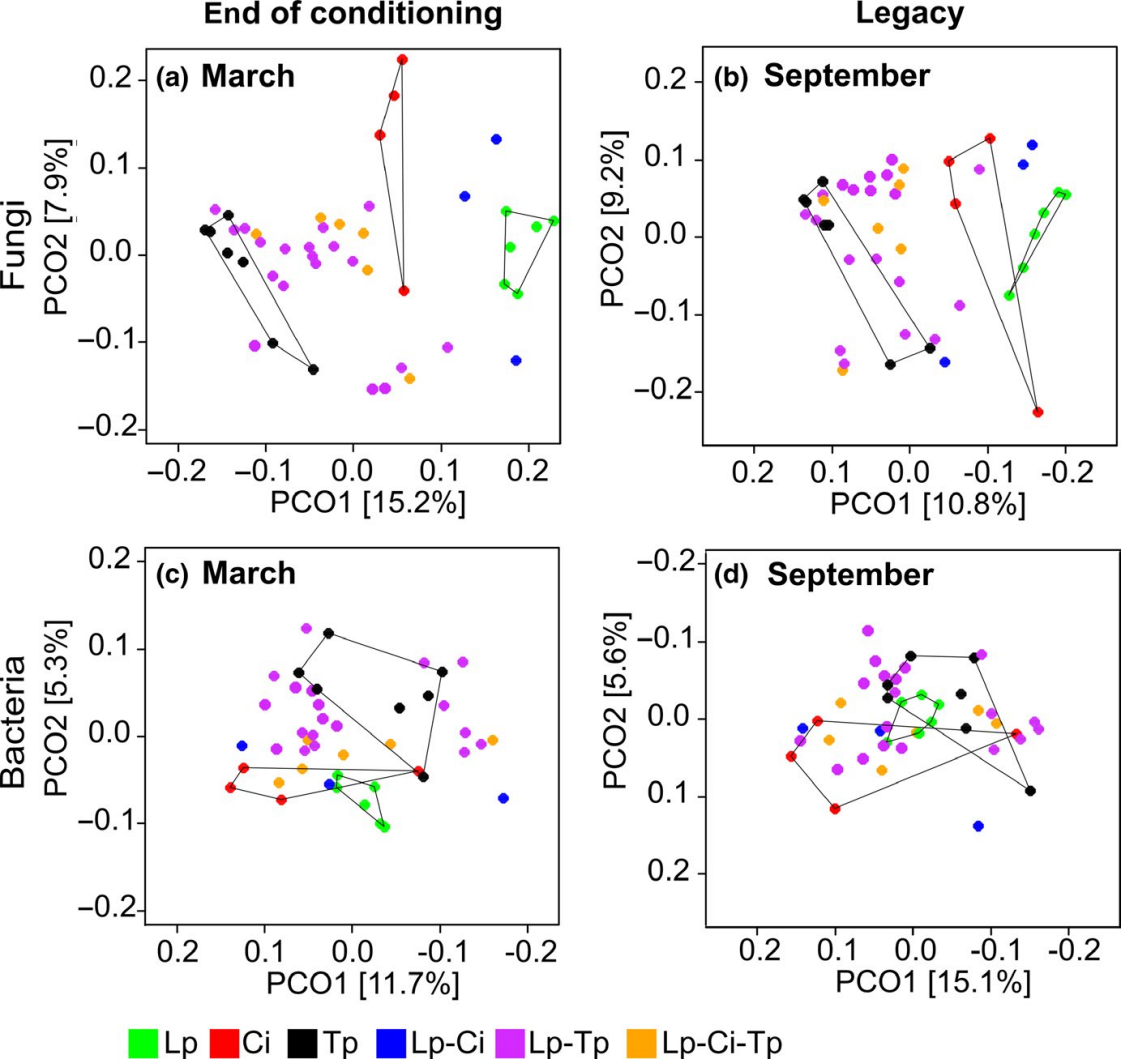
The effect of differing sward types on both fungal (a and b) and bacterial (c and d) community structures as shown by a principal co‐ordinates graph (PCO). This was at the end of the three year conditioning phase (March, a and c) and at the last legacy sampling (September, b and d). For the legacy phase, the different sward types were removed in April and replaced with the following crop *Lolium multiflorum*. Convex hulls were used for all monoculture swards to aid visualization. For completeness, PCO graphs from all four sampling time points are shown in Figure S3. Abbreviations are explained in the legend of Figure 1

For the fungi, 6,584,486 high‐quality ITS sequences were obtained (mean Good's coverage per sample of 1.00) yielding 4,900 fOTUs. At the end of the conditioning phase, the factor "sward type" revealed highly significant differences in fungal community structures (Table 1; PERMANOVA, *p* < .001, Figure 2a). The Lp monoculture harbored a significantly distinct fungal community structure to the other swards (*p* < .01, Table 1), except to the Lp–Ci bi‐species sward (likely due to a lack of statistical power). The centroid of Lp had the greatest distance from Tp (0.341), followed by Ci (0.317), both of which were greater than the distance to the three multi‐species swards (0.211–0.274, Table 1).

**Table 1:** The effect of sward type on both fungal and bacterial community structure as tested by PERMANOVA analysis both at the end of the three‐year conditioning phase (March) and during the legacy samplings (June, August, and September)

	Sward type	End of conditioning	Legacy		
Fungi		March	June	August	September
PERMANOVA					
*F*‐value		2.792	2.488	2.296	1.768
*p‐*value (main)		<.001	<.001	<.001	<.001
*R* ^2^		.264	.242	.227	.185
*p‐*value (pairwise)	Lp ~ Ci	.007	.008	.009	.024
	Lp ~ Tp	.005	.003	.003	.005
	Lp ~ Lp–Ci	.071	.117	.123	.038
	Lp ~ Lp–Tp	.002	.002	.002	.002
	Lp ~ Lp–Ci–Tp	.006	.005	.008	.009
PERMDISP					
*F*‐value		4.198	3.368	2.891	1.049
*p*‐value		.004	.013	.023	.396
Distance of centroids (pairwise)	Lp ~ Ci	0.317	0.293	0.292	0.259
	Lp ~ Tp	0.341	0.311	0.309	0.285
	Lp ~ Lp–Ci	0.211	0.201	0.207	0.252
	Lp ~ Lp–Tp	0.265	0.231	0.232	0.243
	Lp ~ Lp–Ci–Tp	0.274	0.251	0.242	0.271
Bacteria					
PERMANOVA					
*F*‐value		1.303	1.315	1.375	1.206
*p*‐value (main)		.002	.002	.002	.032
*R* ^2^		.143	.144	.15	.134
*p‐*value (pairwise)	Lp ~ Ci	.023	.024	.026	.160
	Lp ~ Tp	.012	.006	.026	.005
	Lp ~ Lp–Ci	.483	.446	.696	.160
	Lp ~ Lp–Tp	.015	.037	.044	.124
	Lp ~ Lp–Ci–Tp	.023	.022	.042	.043
PERMDISP					
*F*‐value		2.023	2.104	3.934	2.299
*p*‐value		.097	.088	.007	.065
Distance of centroids (pairwise)	Lp ~ Ci	0.198	0.198	0.185	0.165
	Lp ~ Tp	0.170	0.160	0.178	0.150
	Lp ~ Lp–Ci	0.170	0.166	0.154	0.164
	Lp ~ Lp–Tp	0.145	0.132	0.133	0.122
	Lp ~ Lp–Ci–Tp	0.155	0.153	0.159	0.148

Note

A homogeneity of dispersion (PERMDISP) test between the different sward types at each sampling time was also performed. Finally, the pairwise centroid distance (Euclidean) of each sward type to Lp at each sampling time is shown. Abbreviations are explained in the legend of Figure 1.

The difference in fungal community structures between the different swards seen at the end of the conditioning phase was retained into each of the legacy samplings, where there was a significant effect of previous "sward type" (June, August and September, *p* < .001, Figure 2b, Figure S3b–d) under the new *L. multiflorum* monoculture. Interestingly, the amount of variance in community structure, explained by the factor "sward type," sequentially declined with time, from *R* ^2^ = .264 at the end of the conditioning phase to *R* ^2^ = .185 at September legacy sampling (Table 1, PERMANOVA).

For the bacteria, a total of 5,603,216 high‐quality 16S rRNA sequences were obtained (Good's coverage per sample of 0.96), yielding 13,539 bOTUs. At the end of the conditioning phase, the community structures were significantly influenced by "sward type" (Table 1, PERMANOVA, *p* = .002, Figure 2c). As with the fungi, the Lp monoculture harbored a significantly distinct soil bacterial community when compared to all other swards, though also with the exception of the Lp–Ci bi‐species sward (Table 1). The centroid of the bacterial community in Lp monoculture soil was most distant from that of the Ci monoculture (0.198), followed by the Tp monoculture (0.170). These distances were equal or greater than the distance between the centroid of the Lp monoculture and each of the three multi‐species swards (Table 1).

A significant effect of previous "sward type" on bacterial community structures did persist into all three legacy sampling time points (Figure 2d, Figure S3f–h). The variance in bacterial community structures explained by "sward type" was lower in September (0.134) than in March (0.143), but there was no sequential reduction seen through June (0.144) and August (0.150, Table 1).

### 3.2 Persistent reduction in fungal OTU richness associated with Tp

**Figure 3:**
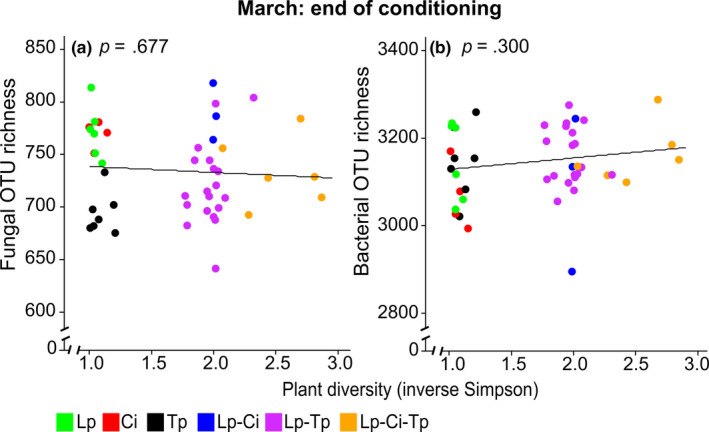
The effect of plant diversity (inverse Simpson index) on fungal (a) and bacterial (b) operational taxonomic units (OTU) richness at the end of the three year conditioning phase (March). Regression lines were included to illustrate the lack of a diversity effect, per se, in OTU richness. Abbreviations are explained in the legend of Figure 1

**Figure 4:**
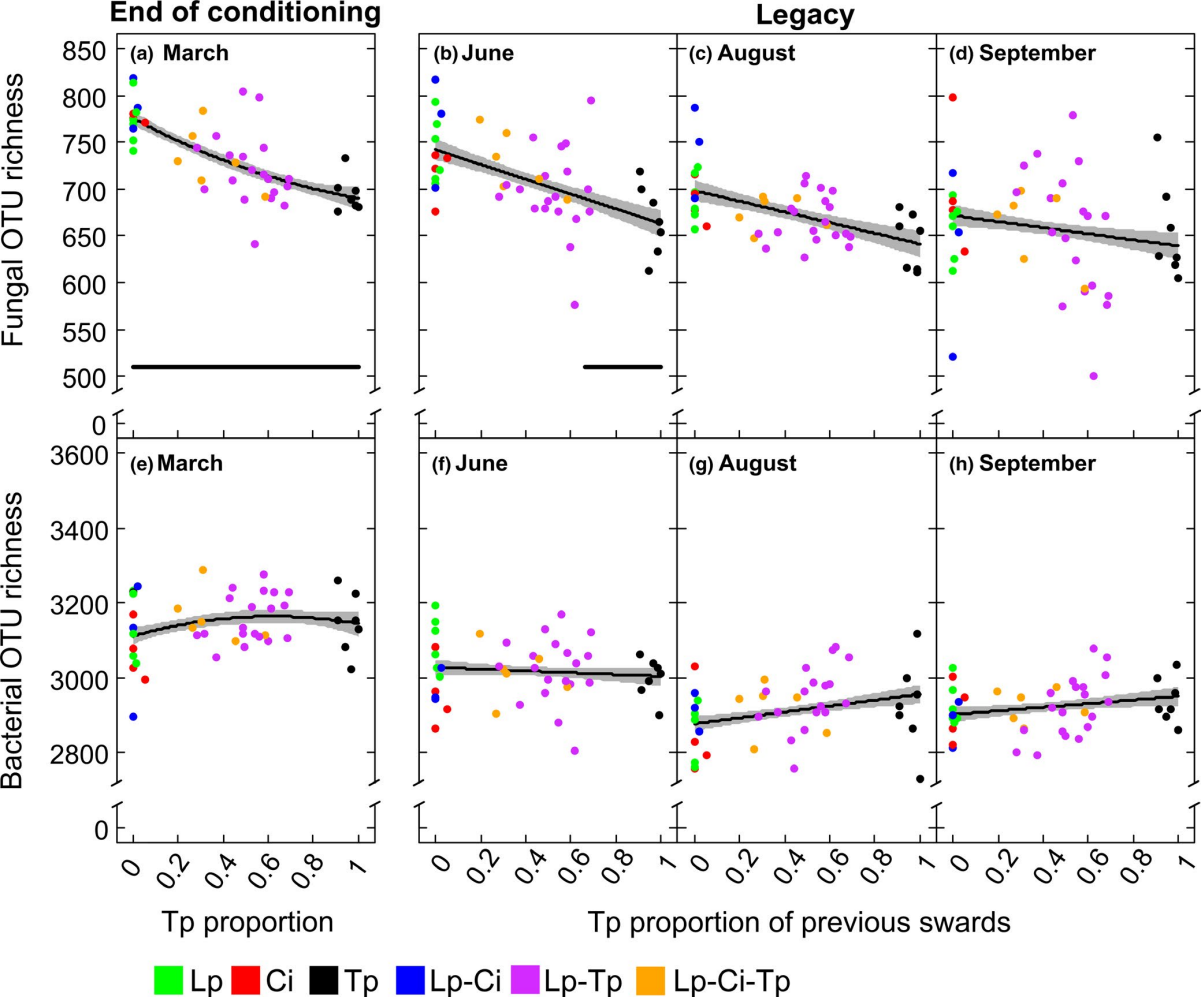
The effect of Tp proportion (0%–100%) on fungal (a–d) and bacterial (e–h) OTU richness at the end of the three‐year conditioning phase in March, and during the legacy samplings in June, August, and September. Displayed are the measured data for each plot (colored dots) and predicted lines (±*SE*, gray shaded). The horizontal bold line in a and b depicts the range of Tp proportions, for which the reduction in fungal OTU richness was significant when compared to the non–Tp containing swards (*p* ≤ .05, no line indicates nonsignificance). Abbreviations are explained in the legend of Figure 1

There was no significant relationship between fungal OTU richness and plant diversity (*R* ^2^ = .004, *p* = .68, Figure 3a). In contrast, at the end of the conditioning phase, there was a highly significant (*p* < .001) effect of "sward type" on fungal OTU richness, with all the legume containing swards (Tp, Lp–Tp and Lp–Ci–Tp) having a significantly reduced fungal OTU richness compared to Lp (Table 2). There was no such difference in either Ci or Lp–Ci compared to Lp, thus making it a Tp/ non–Tp effect. This reduction in fungal OTU richness was shown to be significant over the whole range of Tp proportions, ranging from 0.01%–100% (Figure 4a, *R* ^2^ = .51). A significant reduction in fungal diversity (inverse Simpson (iS) index) was also observed in all sward types, with the exception of the Lp–Ci bi‐species sward, when compared to Lp (Table 2).

**Table 2:** The effect of sward type on both fungal and bacterial OTU richness and inverse Simpson (iS) both at the end of the three year conditioning phase (March) and the three legacy samplings (June, August, and September)

		End of conditioning	Legacy		
Fungi		March	June	August	September
Richness	*F*‐value	7.327	3.834	6.437	0.481
Sward type	*p*‐value	<.001	.006	<.001	.788
	Lp	771.916 (±10.290)	742.228 (±14.466)	690.834 (±10.659)	656.784 (±12.697)
	Ci	769.709 (±6.440) ^ns^	716.564 (±14.007) ^ns^	687.372 (±11.832) ^ns^	699.270 (±34.911)
	Tp	694.013 (±7.454)***	666.947 (±14.105)**	644.420 (±11.251)**	655.225 (±19.896)
	Lp–Ci	789.434 (±15.699) ^ns^	766.061 (±34.223) ^ns^	742.751 (±28.001)*	630.431 (±57.719)
	Lp–Tp	720.223 (±8.940)**	697.090 (±10.714)*	666.479 (±5.969) ^ns^	654.169 (±16.111)
	Lp–Ci–Tp	733.072 (±13.370)*	728.245 (±13.811) ^ns^	674.922 (±7.165) ^ns^	660.695 (±17.035)
iS	*F*‐value	3.516	5.011	0.706	1.271
Sward type	*p*‐value	.010	.001	.622	.296
	Lp	80.790 (±4.252)	78.122 (±4.116)	41.153 (±5.772)	51.645 (±5.564)
	Ci	59.740 (±3.036)*	61.897 (±3.529) ^ns^	55.307 (±8.588)	42.188 (±11.033)
	Tp	60.775 (±1.565)*	53.341 (±4.780)**	44.369 (±4.728)	56.459 (±6.319)
	Lp–Ci	85.994 (±11.711) ^ns^	92.573 (±5.349) ^ns^	55.881 (±6.888)	41.868 (±19.095)
	Lp–Tp	61.002 (±3.494)**	61.443 (±3.641)*	47.536 (±3.413)	55.671 (±4.592)
	Lp–Ci–Tp	58.713 (±8.601)*	64.969 (±5.001) ^ns^	46.834 (±6.765)	36.952 (±5.884)
Bacteria					
Richness	*F*‐value	1.165	1.852	1.044	0.316
Sward type	*p*‐value	.344	.125	.406	.9
	Lp	3,149.722 (±36.656)	3,092.852 (±30.779)	2,848.096 (±29.701)	2,928.064 (±23.354)
	Ci	3,067.062 (±38.431)	2,956.959 (±46.327)	2,851.934 (±61.325)	2,908.193 (±40.149)
	Tp	3,146.394 (±30.507)	2,999.174 (±20.343)	2,926.858 (±45.192)	2,939.769 (±22.936)
	Lp–Ci	3,091.066 (±103.164)	2,972.453 (±26.659)	2,911.409 (±29.586)	2,882.533 (±35.745)
	Lp–Tp	3,159.494 (±14.775)	3,021.783 (±20.344)	2,933.207 (±22.804)	2,921.491 (±18.762)
	Lp–Ci–Tp	3,161.455 (±28.039)	3,012.212 (±29.164)	2,915.722 (±28.891)	2,924.779 (±17.804)
iS	*F*‐value	1.447	1.372	1.402	0.459
Sward type	*p*‐value	.229	.256	.245	.804
	Lp	269.010 (±12.188)	283.211 (±24.290)	211.192 (±34.809)	311.953 (±12.666)
	Ci	333.509 (±31.504)	280.516 (±20.606)	282.803 (±44.444)	340.790 (±23.561)
	Tp	326.245 (±23.049)	237.666 (±19.376)	325.428 (±40.397)	316.280 (±21.231)
	Lp–Ci	304.431 (±64.441)	265.279 (±37.158)	266.092 (±73.915)	350.093 (±15.142)
	Lp–Tp	324.687 (±11.597)	287.643 (±11.076)	305.576 (±19.315)	327.467 (±10.621)
	Lp–Ci–Tp	288.567 (±9.976)	246.318 (±21.379)	294.166 (±11.810)	316.989 (±21.329)

Note

The effect of sward type and their pairwise differences were tested with ANOVA analysis. Corresponding *F‐* and *p‐*values refer to the overall effect, and the significance tests to sward types are against Lp, based on post hoc contrasts. Given are the means for each sward type (±1 *SE*). Abbreviations are explained in the legend of Figure 1. Significance codes: "***" *p* ≤ .001 "**" *p* ≤ .01 "*" *p* ≤ .05 "ns*" p > *.05.

An effect of previous "sward type" on fungal OTU richness was retained into the legacy samplings in June and August (*p* = .006 and *p* < .001, respectively; Table 2), and the values for the previous Tp monoculture were significantly lower than in Lp at both time points (*p* ≤ .01; Table 2). This effect was not sustained into the September sampling (Table 2). For the June legacy sampling, it was shown that fungal OTU richness was still significantly reduced over the previous 66%–100% Tp range when compared to the previous 0% Tp proportion (Figure 4b, marginal and conditional *R* ^2^ were 0.28 and 0.40, respectively). The reduction of fungal OTU richness with increased previous Tp proportion was no longer significant in August and September (Figure 4c,d). A significant legacy effect of previous "sward type" was also seen on fungal diversity (iS index) in June (*p* = .001), where a significant reduction was maintained for the previous Tp and Lp–Tp swards when compared to the previous Lp sward (Table 2).

Like fOTU richness, there was no significant effect of plant diversity on bacterial OTU richness (*R* ^2^ = .16, *p* = .30, Figure 3b). In contrast to fungi, there was also no significant effect of sward type on bacterial OTU richness or iS in March or in any of the legacy samplings (Table 2). There was also no Tp/ non–Tp effect on bacterial OTU richness detected at the end of the conditioning phase (Figure 4e, *R* ^2^ = .07), nor at any of the legacy samplings (Figure 4f–h, marginal *R* ^2^ = .28, conditional *R* ^2^ = .40).

### 3.3 Significant effect of sward type on fungal and bacterial phyla

The fOTUs could be assigned to 11 phyla. Seven of them revealed a total relative sequence abundance of >0.1% in the Lp monoculture at the end of the conditioning phase with the Ascomycota being the most abundant in all sward types (73.5%–79.8%, Table 3). There was a significantly lower mean relative abundance of the fungal phylum Glomeromycota in each of the Tp containing sward types when compared to the Lp monoculture (1.67%), that is, Tp (0.30%), Lp–Tp (0.59%), and Lp–Ci–Tp (0.86%, all *p* ≤ .001; Table 3). Looking at the legacy samplings, a highly significant effect of sward type was retained on the relative abundance of the Glomeromycota in June (*p* < .001, Table S1), with all previous Tp containing sward types having a significantly lower abundance of the Glomeromycota compared to previous Lp. There was no main effect of sward type on the Glomeromycota in either August or September (Table S1). The relative abundance of a third fungal phylum, the Basidiomycota, was also affected by sward type, with it being significantly reduced in Lp–Tp compared to Lp (Table 3).

**Table 3:** The effect of sward type on the relative abundance (%) of the eleven fungal phyla and the major bacterial phyla (cutoff 0.1%) at the end of the three year conditioning phase in March

	*F*‐value	*p*‐value	Sward types					
			Lp	Ci	Tp	Lp–Ci	Lp–Tp	Lp–Ci–Tp
Fungi								
Ascomycota	2.372	.057	75.163 (±1.392)	76.543 (±3.235)	79.828 (±0.846)	73.467 (±1.617)	79.545 (±0.906)	74.535 (±3.294)
Basidiomycota	2.609	.04	16.848 (±1.260)	13.944 (±2.666)^ns^	12.108 (±0.646)^ns^	18.595 (±1.361)^ns^	12.687 (±0.767)*	17.308 (±3.324)^ns^
Glomeromycota	23.930	<.001	1.669 (±0.130)	1.632 (±0.233)^ns^	0.303 (±0.047)***	1.616 (±0.279)^ns^	0.587 (±0.069)***	0.860 (±0.116)***
Mortierellomycota	0.444	.815	1.590 (±0.202)	1.651 (±0.189)	1.916 (±0.087)	1.510 (±0.185)	1.937 (±0.209)	1.782 (±0.214)
Chytridiomycota	1.683	.162	0.992 (±0.276)	1.154 (±0.218)	0.643 (±0.099)	0.664 (±0.088)	0.751 (±0.078)	0.604 (±0.078)
Mucoromycota	1.380	.253	0.498 (±0.087)	1.219 (±0.303)	0.769 (±0.271)	0.687 (±0.249)	0.580 (±0.085)	0.940 (±0.361)
Rozellomycota	1.322	.275	0.107 (±0.025)	0.072 (±0.003)	0.104 (±0.022)	0.116 (±0.022)	0.155 (±0.018)	0.111 (±0.042)
Blastocladiomycota	0.607	.695	0.021 (±0.010)	0.007 (±0.002)	0.037 (±0.025)	0.034 (±0.013)	0.042 (±0.012)	0.048 (±0.013)
Monoblepharomycota	1.648	.170	0.014 (±0.006)	0.020 (±0.008)	0.008 (±0.003)	0.019 (±0.012)	0.011 (±0.002)	0.023 (±0.004)
Olpidiomycota	0.862	.515	0.008 (±0.003)	0.027 (±0.010)	0.011 (±0.004)	0.025 (±0.024)	0.026 (±0.006)	0.020 (±0.006)
Kickxellomycota	0.860	.517	0.003 (±0.002)	0.001 (±0.001)	0.003 (±0.002)	n.d	0.003 (±0.001)	0.005 (±0.003)
Bacteria								
Actinobacteria	0.591	.707	24.587 (±0.984)	26.410 (±1.264)	25.642 (±0.845)	25.530 (±1.179)	25.041 (±0.586)	24.157 (±0.654)
Proteobacteria	0.553	.735	18.144 (±0.272)	17.893 (±0.199)	17.502 (±0.406)	17.520 (±0.445)	18.067 (±0.265)	18.159 (±0.347)
Chloroflexi	0.460	.803	13.612 (±0.622)	13.201 (±1.118)	13.442 (±0.580)	14.314 (±1.198)	13.153 (±0.270)	13.012 (±0.442)
Verrucomicrobia	0.392	.851	13.469 (±0.643)	12.771 (±1.366)	13.532 (±0.712)	13.295 (±1.295)	13.445 (±0.356)	14.244 (±0.300)
Planctomycetes	2.843	.028	11.337 (±0.437)	10.384 (±0.109)*	10.085 (±0.205)**	10.389 (±0.146)*	10.535 (±0.146)*	10.283 (±0.170)**
Acidobacteria	1.122	.365	7.534 (±0.171)	7.372 (±0.440)	7.419 (±0.338)	6.860 (±0.382)	7.627 (±0.216)	8.167 (±0.325)
Firmicutes	1.978	.104	2.716 (±0.150)	2.383 (±0.143)	3.163 (±0.257)	3.000 (±0.265)	2.948 (±0.122)	2.545 (±0.135)
Patescibacteria	0.368	.867	2.248 (±0.068)	2.124 (±0.319)	2.067 (±0.143)	2.354 (±0.457)	2.045 (±0.126)	2.258 (±0.233)
Bacteroidetes	3.134	.018	2.154 (±0.165)	3.450 (±0.339)***	3.013 (±0.199)**	2.826 (±0.461)^ns^	2.973 (±0.131)**	2.912 (±0.165)*
Gemmatimonadetes	5.687	<.001	1.003 (±0.040)	0.953 (±0.047)^ns^	1.170 (±0.038)**	0.975 (±0.044)^ns^	1.136 (±0.022)**	1.106 (±0.033)^ns^
Latescibacteria	1.106	.373	0.652 (±0.039)	0.735 (±0.046)	0.649 (±0.026)	0.592 (±0.030)	0.625 (±0.024)	0.659 (±0.040)
Rokubacteria	1.11	.371	0.593 (±0.049)	0.536 (±0.101)	0.489 (±0.035)	0.467 (±0.043)	0.491 (±0.027)	0.582 (±0.059)
Nitrospirae	2.704	.034	0.386 (±0.018)	0.390 (±0.050)^ns^	0.522 (±0.036)*	0.321 (±0.069)^ns^	0.495 (±0.030)*	0.474 (±0.031)^ns^
Entotheonellaeota	0.478	.79	0.148 (±0.016)	0.141 (±0.021)	0.126 (±0.011)	0.148 (±0.037)	0.130 (±0.008)	0.125 (±0.013)
Elusimicrobia	1.641	.172	0.141 (±0.012)	0.107 (±0.008)	0.139 (±0.009)	0.126 (±0.017)	0.135 (±0.007)	0.160 (±0.018)
Chlamydiae	1.739	.149	0.134 (±0.017)	0.107 (±0.010)	0.147 (±0.013)	0.133 (±0.010)	0.155 (±0.011)	0.110 (±0.014)
Cyanobacteria	1.616	.179	0.101 (±0.011)	0.157 (±0.020)	0.115 (±0.011)	0.119 (±0.024)	0.104 (±0.009)	0.096 (±0.021)

Note

The effect of sward type and their pairwise differences were tested with ANOVA. Corresponding *F‐* and *p*‐values refer to the overall effect, and the significance tests to sward types are against Lp, based on post hoc contrasts. Given are the mean for each sward type (±1 *SE*). Abbreviations are explained in the legend of Figure 1. Significance codes: "***" *p* ≤ .001 "**" *p* ≤ .01 "*" *p* ≤ .05 "ns*" p > *.05. "n.d" signifies no detection.

The bOTUs could be assigned to 37 phyla, 17 of which had a total relative sequence abundance of >0.1% in the Lp monoculture at the end of the conditioning phase. Of these, four phyla significantly responded to sward type (Table 3). The relative abundance of the Planctomycetes was significantly reduced in all sward types compared to Lp, while the opposite was the case for the Bacteroidetes, where each sward type was significantly increased over Lp, with the exception of the Lp–Ci bi‐species sward. The relative abundance of both the Gemmatimonadetes and the Nitrospirae did significantly (*p* ≤ .01 and *p* ≤ .05, respectively) increase in both Tp (1.17 and 0.52%, respectively) and Lp–Tp (1.14 and 0.5%, respectively) over Lp (1 and 0.39%, respectively, all Table 3). In the legacy samplings, the patterns of abundance of these four bacterial phyla seen in March were not retained, with no consistent pattern with previous sward type observed (Table S1). The lesser abundant (<0.1%) bacterial phyla in the different sward types are listed in Table S3 but were excluded from more detailed analyses.

### 3.4 The abundance of the Glomeromycota declined with increased Tp proportion

**Figure 5:**
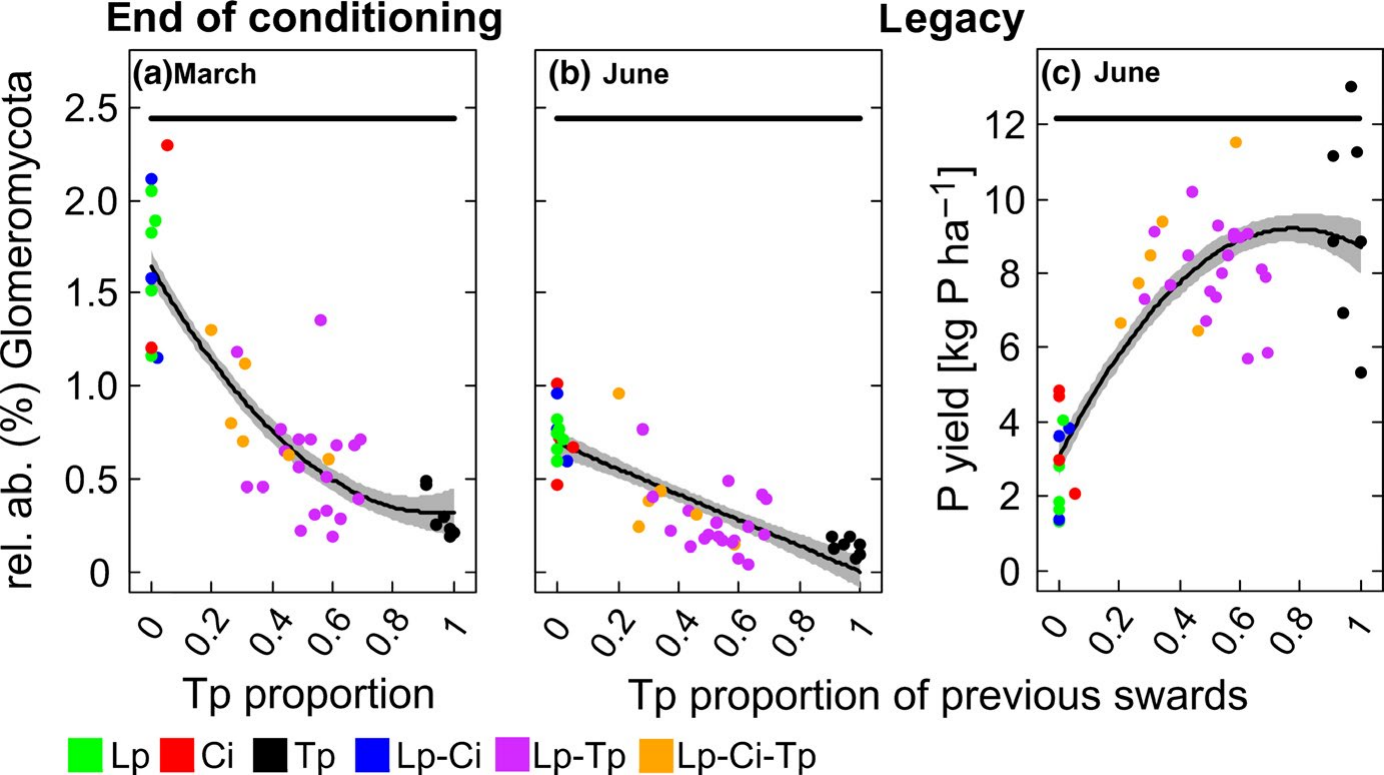
The effect of Tp proportion (0%–100%) on the relative abundance (%, rel. ab.) of the Glomeromycota at the end of the three year conditioning phase in March (a) and at the legacy sampling in June (b), as well as the P yield of the following *Lolium multiflorum* crop harvested in June (c). Displayed are the measured data for each plot (colored dots) and predicted lines (±*SE*, gray shaded). The horizontal bold line in all panels depict the range of Tp proportions for which the relative abundance (%) of the Glomeromycota (a and b) and the *L. multiflorum* P yield was significant when compared to the non–Tp containing swards (*p* ≤ .05). Abbreviations are explained in the legend of Figure 1

As the relative abundance of the Glomeromycota was most clearly responding to Tp/ non–Tp conditions, more detailed analyses were performed on this fungal taxon. At the end of the conditioning phase, the relative abundance of this phylum significantly declined over the entire range of Tp proportions, ranging from 0.01% to 100%, when compared against a 0% Tp proportion (*R* ^2^ = .76, Figure 5a). In the June legacy sampling, there was still a significant reduction over the entire range of previous Tp proportions (0.01%–100%, Figure 5b, marginal and conditional *R* ^2^ were 0.46 and 0.51, respectively). There was, however, no longer an effect of increasing previous Tp proportion on the relative abundance of this phylum in the August or September legacy samplings (Figure S4c,d). Taking a deeper look into taxonomy, fourteen named genera were distinguished within the phylum Glomeromycota at the end of the conditioning phase in March and nine of them revealed a significant response to sward type (Table S3). Of these, five genera, that is, *Claroideoglomus*, *Glomus*, *Dominikia*, *Archaeospora,* and *Diversispora*, had a lower relative abundance in each of the Tp containing swards when compared against Lp. Despite the decline in abundance of the Glomeromycota, seen at the end of the conditioning phase and at the first legacy sampling in June, there was a clear and significant increase, over the entire range of previous Tp proportions, in the P yield of the following *L. multiflorum* crop at the June harvest (Figure 5c). This increased from 3.031 (±0.471) kg P/ha in the previous 0% Tp proportion to 8.701 (±0.710) kg P/ha in the previous Tp monoculture.

### 3.5 Indicator fungal and bacterial OTUs associate with different plant monocultures

At the end of the conditioning phase, indicator species analysis on the three different monocultures (Lp, Ci and Tp) revealed that 289 fOTUs could be designated indicator OTUs for plant species (i.e., 173 OTU unique to each monoculture and 116 shared between two, Table 4, visualized in Figure S5, left), with 173 fOTUs being uniquely associated to one monoculture (81 to Lp, 55 to Ci and 37 to Tp). These fOTUs originated from a broad range of taxonomic groups (Table S4). A portion of the indicator fOTUs, which were found to be associated with the swards at the end of the conditioning phase, were also detected as such in one or more of the legacy samplings (Table 4). There was also a sequential reduction in the total number of indicator fOTUs observed for each of the three legacy samplings. Of the 289 fOTUs found at the end of the conditioning phase, 117 were also detected in June, 100 in August, and 66 in September. Each of the monocultures did, however, have a set of associated indicator fOTUs, which were detected in each of the legacy samplings; 8 for Lp, 2 for Ci, and 7 for Tp (Table S4).

**Table 4:** The effect of the monoculture swards on the number of indicator (IndVal ≥ 0.8, *p‐*value ≤ .05) fungal and bacterial OTUs (fOTU and bOTU, respectively) detected at the end of the three year conditioning phase (March) and subsequently during the three legacy samplings (June, August, and September)

	End of conditioning	Legacy		
Fungi	March	June	August	September
Total	289	117	100	66
Unique Lp	81	24	23	11
Unique Ci	55	14	10	3
Unique Tp	37	18	15	11
Bacteria				
Total	342	68	73	52
Unique Lp	47	8	5	4
Unique Ci	67	9	8	2
Unique Tp	41	7	11	8

Note

Shown are the total indicator fOTUs and bOTUs detected (i.e., the sum of OTUs found in each monoculture type as well as those shared between two monoculture types) and the number unique to each of the three monocultures. Abbreviations are explained in the legend of Figure 1. A graphical representation of the distribution of indicator f and bOTUs between the three monoculture types which were detected in March is shown in Figure S3.

There were also 342 indicator bOTUs associated to plant species, 155 of which were unique to one monoculture and 187 shared between two (Table 4, as well as Figure S5, right). Again, there was a portion of the indicator bOTUs detected at the end of the conditioning phase which was also found in the following legacy samplings. Unlike with the fungi, however, there was no sequential reduction in the total number of indicator bOTUs detected (Table 4). Additionally, only a very small number of unique indicator bOTUs detected at the end of the conditioning phase were also found in each of the three legacy samplings (1 in Lp, 0 in Ci, and 3 in Tp; Table S5).

## 4 DISCUSSION

In this field experiment, we investigated the persistent effects that different plant species, when grown in monocultures and mixtures, had on soil microbial communities using a metabarcoding approach. It demonstrated that all three species grown in monoculture had highly significant effects on all examined aspects (community structure, microbial diversity analyzed as richness or iS index, the relative abundance of individual phyla and indicator OTUs) of both fungal and bacterial communities, with the effect being stronger in the former. Interestingly, in the multi‐species swards, all aspects indicated mainly a proportional blending of the individual plant species effects observed in the monocultures (identity effects), rather than an additional diversity effect derived from plant species interactions. Even after the different swards had been removed and a following crop of *L. multiflorum* had been established, significant and sustained effects (legacy effects) of the previous plant species were seen in multiple aspects of the microbial communities, namely community structure, OTU richness, abundance of AM fungi, and the persistence of indicator OTUs.

### 4.1 Plant species identity is a strong driver of microbial community structure

At the end of a three year conditioning phase, this study demonstrated a highly significant influence of plant species identity on both fungal and bacterial community structures in soil. Both Ci and Tp monocultures harbored significantly different fungal and bacterial communities when compared to the Lp monoculture. This finding is in line with previous field based studies, which reported differences in microbial community structures between agronomic grassland species (e.g., Detheridge et al., 2016; Ladygina & Hedlund, 2010). The effect of plant species identity on microbial community structures could also be seen at the indicator OTU level, with a significant number of fOTUs and bOTUs being uniquely associated to each monoculture. Furthermore, the distance between fungal community centroids did show that Tp was most different from Lp, suggesting that the physiological differences between these two plant species, and possibly the release of symbiotically‐fixed N_2_ from Tp (Hammelehle et al., 2018; Oberson et al., 2013), may be an important driver of fungal community structure. Symbiotic N_2_‐fixation and the resulting sloughing of N‐rich exudates and litter by Tp will lower the C/N ratio of SOM, with the N concentration of Tp shoots (~34.1 g of N/kg DW) reported to be much greater than that of Lp (~27.5 g of N/kg DW), under a comparable cropping system (Nyfeler et al., 2011). Differences in SOM chemistry has been shown to be an important driver of soil microbial communities (Fierer et al., 2007). In addition to root exudate quality, the quantity of exudates entering the soil matrix may also differ under the three different plant species. The root biomass of Lp (4.26 g/dm^3^) has been shown to be four times greater than that of Tp (1.28 g/dm^3^), in the first 10 cm of the soil profile in the conditioning phase of this experimental system (Hofer et al., 2017). This may result in differing quantities of C being deposited into the soil matrix through the sloughing of root residues and rhizodeposits. Similar processes are likely the reason for the structural changes in the soil microbial community between Lp and Ci, with the former having twice the root biomass of Ci (2.11 g/dm^3^, Hofer et al., 2017). Their rooting system architectures also sharply contrast (i.e., shallow and filamentous vs. deep tap root), and this may result in differing C‐deposition patterns (Black et al., 2009; Brown et al., 2003). Additionally, there are indications that the root exudates of Ci (such as sesquiterpene lactones) can exert antimicrobial activities (Foster, Cassida, & Sanderson, 2011; Padilla‐Gonzalez, dos Santos, & Da Costa, 2016), which could impact microbial community structure.

A plant species identity effect was also seen on the abundance of the fungal phylum Glomeromycota (discussed in greater detail in section 4.2), as well as various bacterial phyla. The most interesting of which was the Nitrospirae, which was higher in the Tp monoculture compared to the Lp monoculture. This phylum is involved in nitrite oxidation in a wide range of habitats (Lücker et al., 2010) and has been reported as being enhanced by legumes in comparison with grasses in mesocosms (Zhou et al., 2017). The response in the abundance of this phylum is likely a consequence of the effect Tp has on the soil N cycle, with its high N inputs (Hammelehle et al., 2018) which can be transferred through soil processes to the companion grass of the sward (Oberson et al., 2013). Both Ci and Tp were also found to have significantly different abundances of the phyla Planctomycetes and Bacteroidetes compared to Lp. This may be a consequence of variations in SOM and lability under the different plant species, as a consequence of the aforementioned differences in rooting morphology and root exudate chemistry in both these plant species, compared to Lp, as such differences have been reported to influence both the Planctomycetes (Buckley et al., 2006) and the Bacteroidetes (Fierer et al., 2007). The alterations in SOM chemistry induced by a legume may also alter the size distribution of soil aggregate fractions (Zhou et al., 2020). Such a process may explain the difference in the abundance of the Gemmatimonadetes between Tp and Lp, as this phylum has been reported to be quite abundant in the inner micro‐aggregate (Mummey, Holben, Six, & Stahl, 2006).

### 4.2 Plant diversity was not an important driver of soil microbial communities

In addition to looking at monocultures, we also focused on the influence that mixing these species in various multi‐species swards, over a wide range of relative abundances, had on microbial community structure. Plant diversity, per se, had no significant effect, however, on either fungal or bacterial OTU richness in this study (Figure 3). Both these variables did not change with increasing plant diversity levels. If diversity had an impact on OTU richness, the regression line would have been sloped (Kirwan et al., 2007), as has been shown for expected farmer revenues from grasslands due to forage yields, which increased with plant diversity for a multisite experiment in intensively managed fertile grasslands comparable to our experimental system (Schaub, Buchmann, Lüscher, & Finger, 2020) as well as in an experiment comparing different management intensity levels and plant species richness of up to 60 species (Schaub, Finger, et al., 2020).

There was also no indication of a diversity effect in soil microbial community structure either. The centroid distance to Lp is greater between the other two monocultures than between the multi‐species swards, for both fungal and bacterial community structure (with the exception being the distance of Tp and Lp–Ci to Lp in the later, which were the same). This would suggest a blending of microbial community structure in mixture swards, rather than there being an additive diversity effect.

Interestingly, both fungal OTU richness and the relative abundance of the fungal phylum Glomeromycota, whose members are capable of forming arbuscular mycorrhizal (AM) associations with plants (Scheublin, van Logtestijn, & van Der Heijden, 2007), displayed a significant continual decline from 0.01% to 100% Tp proportion. Nonlinear responses to increased Tp proportion would indicate diversity effects (Kirwan et al., 2009; Fox et al., 2020 and conceptualized in Figure 1a), but this was generally not observed in our study. This would indicate that changes between plant species identity is a stronger determinant of fungal OTU richness than plant species diversity. This decline is likely the consequence of continual lower root biomass with increasing Tp proportion, as both Lp and Ci have more root biomass in comparison (Hofer et al., 2017; Ryan et al., 2000). This would indicate that the root infection area in Tp is lower, as well as the rhizodeposition rate, leading to conditions which reduce the relative abundance of Glomeromycota and other fungal species. The gain in soil N with increasing Tp proportion may also be playing a role, as increased soil nutrient availability may lower the abundance of mycorrhizal fungi (Treseder, 2004).

The lack of a plant diversity effect on the soil microbiome contrasts with the highly significant diversity effects on aboveground plant measures such as yield (+77%, Finn et al., 2013; +106% Nyfeler et al., 2009) and biomass yield of a following crop (+132%, Fox et al., 2020; and P yield + 187%, Figure 5c, both for the June harvest). Such large effect sizes, derived from plant species interactions (Figure 1a), suggest a strong influence of plant diversity on ecosystem functioning, due to plant residue input into soil and consequential changes in nutrient cycling and nutrient uptake. Therefore, it is difficult to explain why the soil microbiome responded principally to plant species identity, but not diversity. Nevertheless, it was also reported from another grassland experiment that plant functional group identity (particularly legumes) does have a larger influence on microbial community structure than plant species richness (Dassen et al., 2017). In our study, this stronger plant species identity effect on soil microbial communities, which likely reflects the differing functionalities of the plant species and might represent the mechanism to induce changes in soil physicochemical conditions, is discussed in section 4.1 and suggested in Dassen et al. (2017).

### 4.3 Persistent effect of previous sward type on fungal and bacterial community structure

The significant effects of sward type when the swards were still present (as described above) persisted over the three legacy sampling periods (June, August and September), with the effect declining over time. This is in spite of the observation that temporal progression over a growing season has been shown to be a strong driver of both bacterial and fungal community structure in temperate grasslands (Fox, Ikoyi, Creamer, Lanigan, & Schmalenberger, 2017). Legacy effects of previous plant species have previously been demonstrated at various soil trophic levels (Crotty et al., 2016; Detheridge et al., 2016), with the results presented herein demonstrating a legacy effect not just of previous plant monocultures, but also of multi‐species swards.

The exact mechanism of the plant‐sward mediated legacy effect on soil microbial community structure seen in this study is not known. We do, however, put forward that in the system studied, N was the driving nutrient of this Tp induced legacy effect because, firstly, P content of *L. multiflorum* at the 100% Tp proportion at the June harvest was at a level not considered limiting to plant growth (4.17 ± 0.19 g/kg DM, Almeida, Lüscher, Frehner, Oberson, & Nösberger, 1999; Liebisch et al., 2013). Secondly, the content of available P in the soil of our plots was high (2.29 mg P/kg soil), a value interpreted as a stock of available P, allowing the reduction of P‐fertilizer input even under intensive agricultural production (Flisch et al., 2017). As a consequence, both the P yield (from 3.03 ± 0.47 to 8.70 ± 0.71 kg P/ha) and the DM yield (from 0.60 ± 0.09 to 2.09 ± 0.13 t/ha) increased at the legacy harvest in June with increased Tp proportion, from 0% to 100%, respectively. This was the case despite a corresponding legacy reduction in the abundance of AM fungi, further indicating that P was not limiting in this system. The persistence of N‐rich plant residues, particularly plant litter, stubble, and root biomass, in terms of both quantity and quality is likely a strong driver of the observed legacy effect, as the effect must be soil transferred due to the removal of the previous swards. The retention of such plant residues in the soil matrix may facilitate the persistence of microbial groups, which are best adapted to decompose these under the given conditions (Allison et al., 2013). In the system reported here, such a process may underlie a strong, soil‐transferred, predominantly Tp‐related legacy effect on *L. multiflorum* (Fox et al., 2020, Figure 1a). Here, the legacy signature of the microbiome (i.e., persistent indicator fOTUs and bOTUs) may break down these N‐rich residues and SOM associated with the previous Tp sward, making this essential macronutrient bioavailable. Soil‐transferred legacy effects are most likely the consequence of a number of interconnected processes acting in a synergistic manner (Anderson, 2011), with the nature of such processes differing depending on various factors, such as the previous plant, soil type, and climatic conditions. Such legacy effects likely have broader ecological implications as well. Plant legacy effects (caused by plant senescence and death, herbivore grazing, etc.) on the microbiome may influence plant–plant competition (through the mobilization of nutrients from residues of the previous plant), plant establishment and succession as well as overlying plant composition (Kardol et al., 2013).

These results highlight that plant species identity can be a strong driver of soil microbial community structures, likely as a result of the changes in soil physicochemical parameters induced by the differing physiologies of the plant species employed (i.e., symbiotic N_2_‐fixation, differing root biomass, and structures). They also demonstrate that plant diversity had little effect on the microbiome, even though one could have been expected due to the strong diversity effects which have been demonstrated on aboveground plant performance. Finally, the study highlights the critical role the microbiome may play in the establishment and agronomic performance of a following crop in a rotational system, and is an important contribution to the more comprehensive understanding of rotational cropping systems, and of their role in agronomic sustainability.

## CONFLICT OF INTEREST

The authors declare that they have no conflict of interest.

## AUTHOR CONTRIBUTION

**Aaron Fox:** Data curation (lead); Formal analysis (lead); Writing‐original draft (lead). **Franco Widmer:** Conceptualization (equal); Investigation (equal); Methodology (equal); Project administration (equal); Supervision (equal); Visualization (equal); Writing‐original draft (supporting). **Andreas Lüscher:** Conceptualization (equal); Funding acquisition (lead); Investigation (equal); Methodology (equal); Project administration (equal); Visualization (equal); Writing‐original draft (supporting).

## ETHICAL APPROVAL

The work described here did not require ethics approval.

## Supporting information

Supinfo

## Data Availability

DNA sequences were deposited in the NCBI SRA archive with the project number: PRJNA633031. Additional supporting data associated with this article can be found in the Supporting information at the bottom of this article.
