# Supplementary material for: Plant species identity drives soil microbial community structures that persist under a following crop
Source: Ecol Evol. 2020 Jul 23;10(16):8652–68. doi: 10.1002/ece3.6560 (PMC7452769; doi:10.1002/ece3.6560)
Supplement: Supplementary file 1 — Supinfo [file ECE3-10-8652-s001.docx]

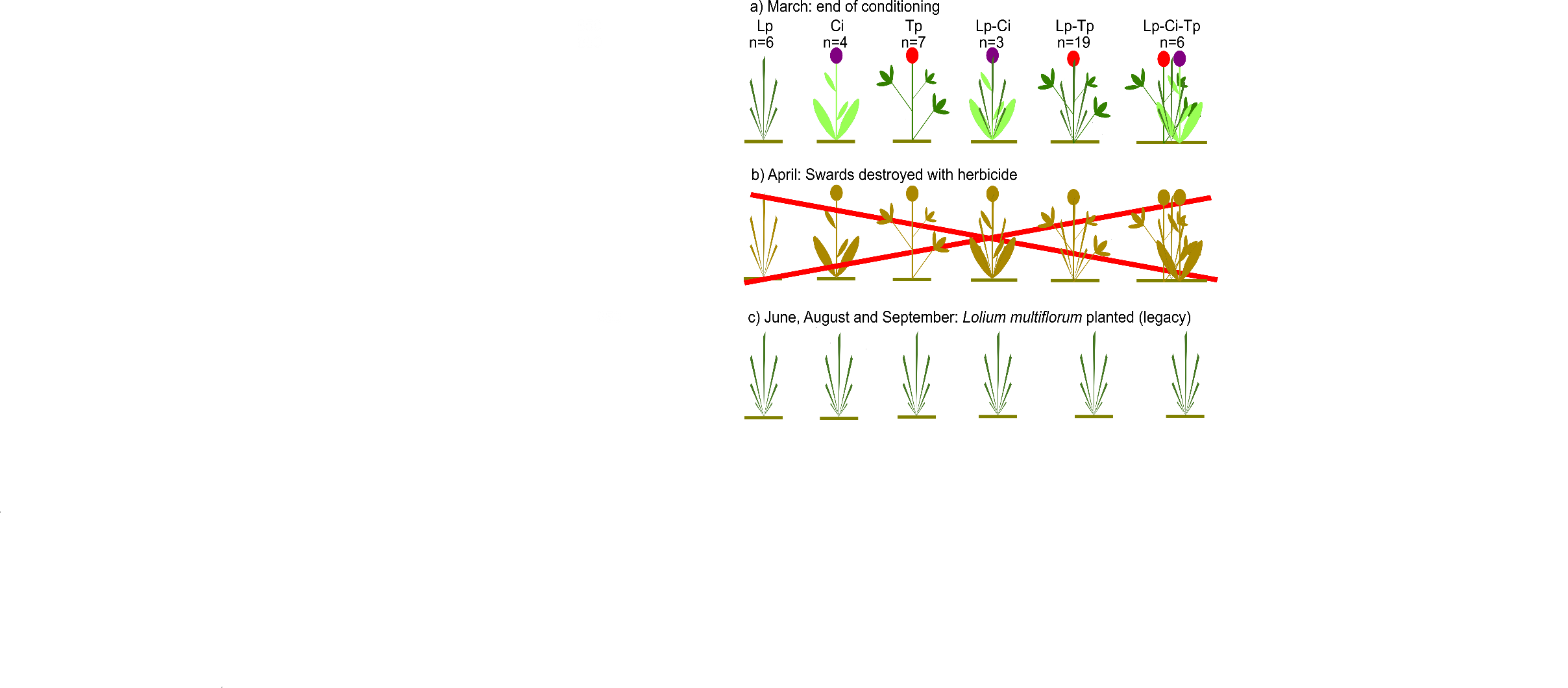


Figure S1: Schematic overview of the experimental setup used in this study. Panel a represents the end of the three year conditioning phase in March 2015, when the six different sward types were still present. In April (panel b), the conditioning phase was terminated with the application of a herbicide and sowing of the following crop of *Lolium multiflorum*. The sampling points in June, August and September (panel c) represent the ‘legacy’ samplings for assessing the lasting effects of the sward type based conditioning. Abbreviations are explained in the legend of Figure 1.


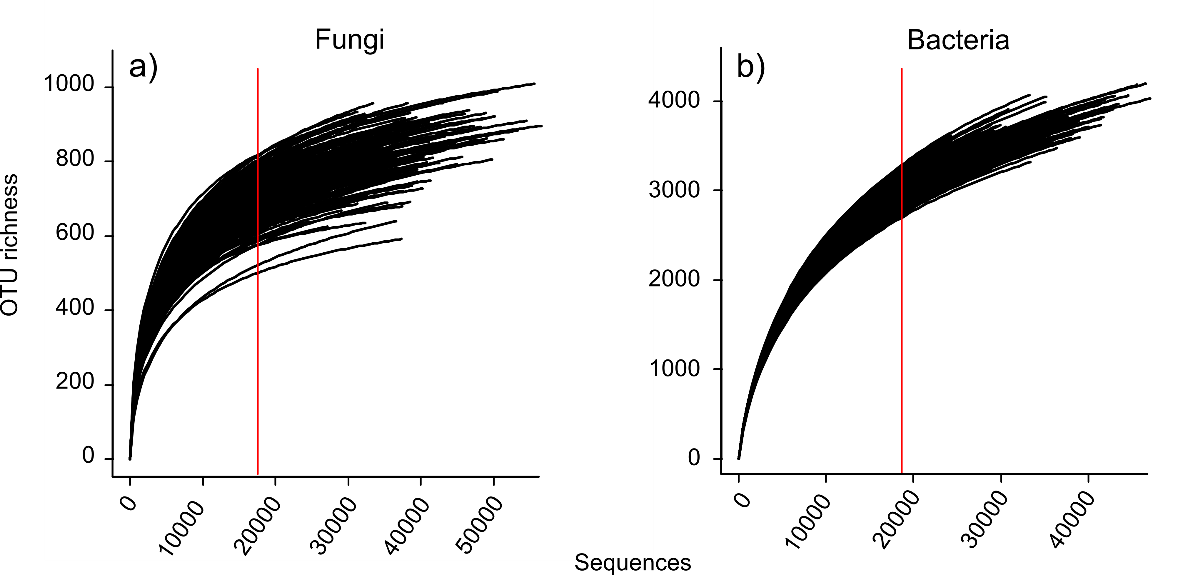


Figure S2: Rarefaction analysis displaying sequencing depth and OTU-saturation in both fungal (panel a) and bacterial (panel b) communities. The red lines indicate the number of high-quality sequences obtained from the sample with the lowest sequencing depth (i.e., 17,542 sequences for fungi and 18,655 sequences for bacteria).


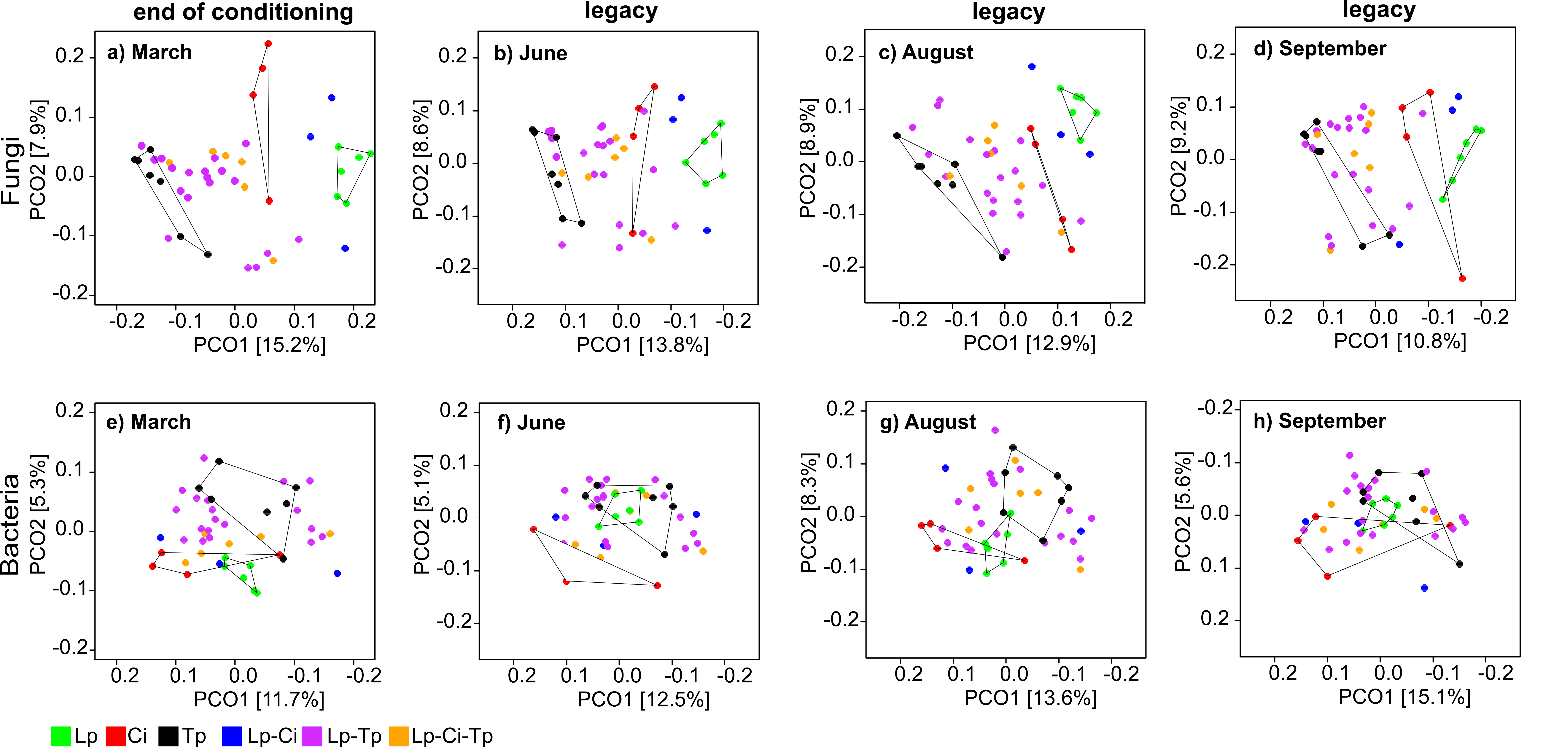


Figure S3: Principal co-ordinates graph (PCO) of the effect of differing sward types had on both fungal (top) and bacterial (bottom) community structure. This was both at the end of the three year conditioning phase (March, panels a and e) and during the legacy samplings (June, August and September, panels b, c, d, f, g and h). Hulls were drawn around all monoculture plots to aid visualization. Abbreviations are explained in the legend of Figure 1.


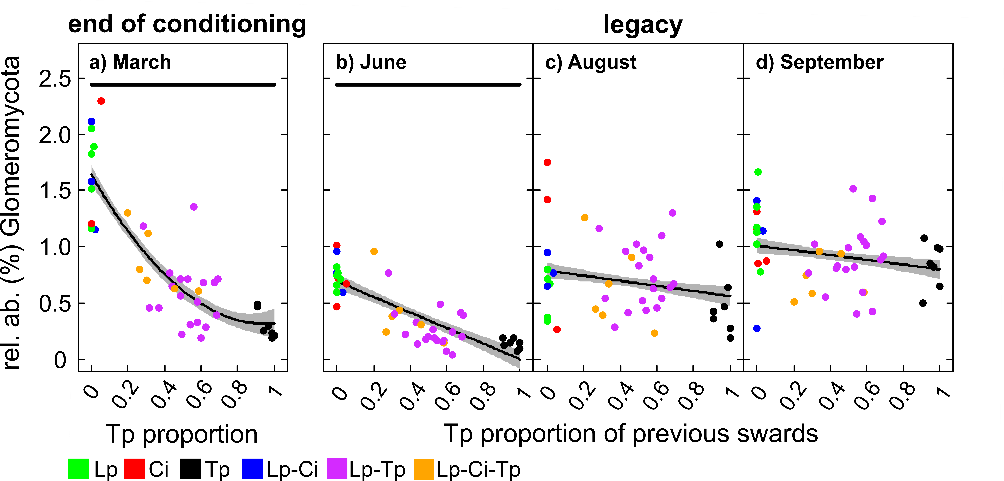


Figure S4: The effect of Tp proportion on the relative abundance (%, rel. ab.) of the Glomeromycota at the end of the three year conditioning phase (March, panel a) and during the legacy samplings (June, August and September, panels b - d). Displays are the measured data (shown as dots) and predicted lines (± SE, grey shaded). The horizontal bold line in panels a and b covers the range of Tp proportions for which the relative abundance of the Glomeromycota is significant when compared to 0% Tp presence (*P* ≤ 0.05, no line indicates non-significance). Abbreviations are explained in the legend of Figure 1.


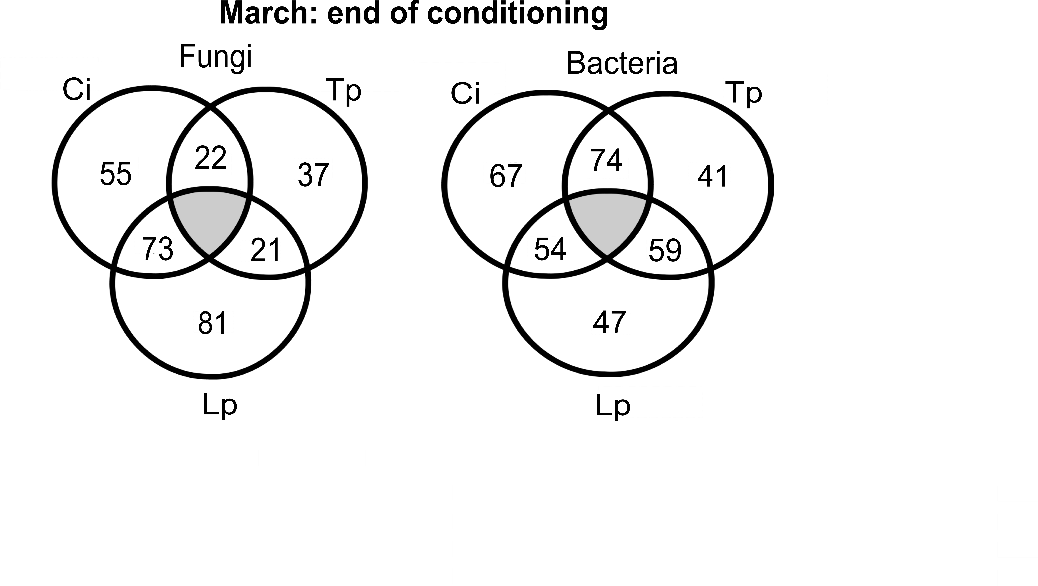


Figure S5: Distribution of fungal (panel a) and bacterial (panel b) indicator OTUs (IndVal ≥ 0.8, *P* – value ≤ 0.05) among the different monoculture sward types at the end of the three year conditioning phase (March). Abbreviations are explained in the legend of Figure 1.

Table S1: The legacy effect of sward type on the relative abundance (%) of both the fungal and bacterial phyla, which displayed a significant response to sward type at the end of the three year conditioning phase (March, see Table 3), during the legacy samplings (June, August and September). Given are the mean (± 1 SE) for each sward type. The effect of sward type and their differences was tested with ANOVA. Corresponding *F* – and *P* – values refer to the overall effect, and the significance tests to sward types are against Lp, based on post-hoc contrasts. Phyla ordered by % relative abundance in the Lp monoculture (highest to lowest). Abbreviations are explained in the legend of Figure 1. Significance codes: ‘***’ *P* **≤** 0.001 ‘**’ *P* **≤** 0.01 ‘*’ *P* **≤** 0.05 ‘ns*’ P >* 0.05.

|  |  |  |  |  |  | Sward type |  |  |  |
| --- | --- | --- | --- | --- | --- | --- | --- | --- | --- |
| Fungi | Sampling | *F* - value | *P* - value | Lp | Ci | Tp | Lp–Ci | Lp–Tp | Lp–Ci–Tp |
| Basidiomycota | June | 2.714 | *P* = 0.034 | 13.249 (±0.528) | 13.559 (±0.841)^ns^ | 10.599 (±0.664)* | 14.768 (±1.225)^ns^ | 11.204 (±0.555)^ns^ | 13.015 (±1.441)^ns^ |
|  | August | 1.505 | *P* = 0.211 | 12.084 (±0.837) | 11.685 (±3.033) | 11.323 (±0.658) | 13.323 (±1.145) | 12.641 (±0.858) | 17.654 (±3.840) |
|  | September | 6.284 | *P* < 0.001 | 17.422 (±1.212) | 14.615 (±1.430)^ns^ | 14.703 (±1.240)^ns^ | 31.063 (±8.389)*** | 15.047 (±0.831)^ns^ | 17.772 (±1.915)^ns^ |
| Glomeromycota | June | 14.69 | *P* < 0.001 | 0.717 (±0.033) | 0.718 (±0.113)^ns^ | 0.137 (±0.016)*** | 0.776 (±0.104)^ns^ | 0.264 (±0.039)*** | 0.413 (±0.118)** |
|  | August | 1.205 | *P* = 0.325 | 0.601 (±0.080) | 0.950 (±0.371) | 0.484 (±0.104) | 0.789 (±0.084) | 0.741 (±0.066) | 0.651 (±0.155) |
|  | September | 1.841 | *P* = 0.127 | 1.187 (±0.123) | 1.050 (±0.115) | 0.838 (±0.077) | 0.939 (±0.342) | 0.901 (±0.068) | 0.721 (±0.078) |
| Bacteria |  |  |  |  |  |  |  |  |  |
| Planctomycetes | June | 1.143 | *P* = 0.354 | 10.507 (±0.394) | 10.520 (±0.281) | 10.067 (±0.294) | 9.966 (±0.244) | 10.328 (±0.135) | 9.799 (±0.194) |
|  | August | 3.819 | *P* = 0.007 | 11.232 (±0.505) | 10.813 (±0.201)^ns^ | 9.281 (±0.365)*** | 9.986 (±0.292)^ns^ | 10.144 (±0.231)* | 9.466 (±0.259)** |
|  | September | 1.623 | *P* = 0.177 | 9.256 (±0.264) | 9.531 (±0.217) | 8.838 (±0.165) | 9.112 (±0.351) | 9.017 (±0.121) | 8.716 (±0.176) |
| Bacteroidetes | June | 1.498 | *P* = 0.213 | 2.438 (±0.068) | 2.660 (±0.088) | 2.608 (±0.088) | 2.616 (±0.146) | 2.627 (±0.078) | 2.895 (±0.141) |
|  | August | 1.732 | *P* = 0.150 | 2.223 (±0.119) | 2.357 (±0.107) | 2.894 (±0.256) | 2.636 (±0.272) | 2.559 (±0.089) | 2.517 (±0.180) |
|  | September | 0.937 | *P* = 0.468 | 2.262 (±0.106) | 2.439 (±0.178) | 2.746 (±0.260) | 2.864 (±0.496) | 2.585 (±0.119) | 2.852 (±0.307) |
| Gemmatimonadetes | June | 3.109 | *P* = 0.019 | 1.231 (±0.066) | 1.058 (±0.060)^ns^ | 1.391 (±0.045)^ns^ | 1.124 (±0.071)^ns^ | 1.338 (±0.044)^ns^ | 1.251 (±0.070)^ns^ |
|  | August | 4.705 | *P* = 0.002 | 1.015 (±0.062) | 0.960 (±0.020)^ns^ | 1.207 (±0.047)* | 0.967 (±0.093)^ns^ | 1.200 (±0.036)** | 1.042 (±0.035)^ns^ |
|  | September | 4.661 | *P* = 0.002 | 1.033 (±0.061) | 1.060 (±0.090)^ns^ | 1.281 (±0.046)** | 0.921 (±0.068)^ns^ | 1.182 (±0.034)^*^ | 1.043 (±0.042)^ns^ |
| Nitrospirae | June | 5.421 | *P* < 0.001 | 0.552 (±0.046) | 0.471 (±0.042)^ns^ | 0.733 (±0.051)** | 0.499 (±0.024)^ns^ | 0.629 (±0.022)^ns^ | 0.567 (±0.017)^ns^ |
|  | August | 0.866 | *P* = 0.513 | 0.432 (±0.046) | 0.470 (±0.059) | 0.424 (±0.052) | 0.364 (±0.065) | 0.495 (±0.035) | 0.409 (±0.040) |
|  | September | 5.418 | *P* < 0.001 | 0.360 (±0.019) | 0.370 (±0.021)^ns^ | 0.545 (±0.046)*** | 0.315 (±0.007)^ns^ | 0.470 (±0.020)** | 0.437 (±0.039)^ns^ |

Table S2: The effect of sward type on the relative abundance (%) of the lesser abundant bacterial phyla (> 0.1%) at the end of the three year conditioning phase (March). The effect of sward type and their differences was tested with ANOVA. Corresponding *F* – and *P* – values refer to the overall effect, and the significance tests to sward types are against Lp, based on post-hoc contrasts. Given are the mean for each sward type (± 1 SE). Phyla ordered by % relative abundance in the Lp sward (highest to lowest). Abbreviations are explained in the legend of Figure 1. Significance codes: ‘***’ *P* **≤** 0.001 ‘**’ *P* **≤** 0.01 ‘*’ *P* **≤** 0.05 ‘ns*’ P >* 0.05. ‘n.d’ signifies no detection.

|  |  |  |  |  | Sward type |  |  |  |
| --- | --- | --- | --- | --- | --- | --- | --- | --- |
| Bacteria | *F* - value | *P* - value | Lp | Ci | Tp | Lp–Ci | Lp–Tp | Lp–Ci–Tp |
| Fibrobacteres | 4.353 | *P* = 0.003 | 0.0971 (±0.0056) | 0.0546 (±0.0156)* | 0.0649 (±0.0033)* | 0.1363 (±0.0218) ^ns^ | 0.0954 (±0.0076) ^ns^ | 0.0901 (±0.0103) ^ns^ |
| Armatimonadetes | 1.955 | *P* = 0.107 | 0.0664 (±0.0044) | 0.0548 (±0.0074) | 0.0473 (±0.0031) | 0.0619 (±0.0154) | 0.0679 (±0.0045) | 0.0551 (±0.0056) |
| BRC1 | 1.029 | *P* = 0.414 | 0.0585 (±0.0072) | 0.0548 (±0.0048) | 0.0700 (±0.0072) | 0.0605 (±0.0103) | 0.0743 (±0.0058) | 0.0608 (±0.0089) |
| Omnitrophicaeota | 2.032 | *P* = 0.095 | 0.0280 (±0.0031) | 0.0224 (±0.0065) | 0.0186 (±0.0031) | 0.0207 (±0.0026) | 0.0185 (±0.0025) | 0.0333 (±0.0072) |
| FCPU426 | 0.464 | *P* = 0.801 | 0.0217 (±0.0038) | 0.0179 (±0.0069) | 0.0270 (±0.0069) | 0.0137 (±0.0064) | 0.0227 (±0.0028) | 0.0241 (±0.0083) |
| Dependentiae | 2.024 | *P* = 0.097 | 0.0187 (±0.0031) | 0.0196 (±0.0052) | 0.0091 (±0.0023) | 0.0277 (±0.0079) | 0.0239 (±0.0030) | 0.0182 (±0.0051) |
| WS4 | 0.414 | *P* = 0.836 | 0.0163 (±0.0030) | 0.0140 (±0.0015) | 0.0186 (±0.0046) | 0.0147 (±0.0074) | 0.0165 (±0.0029) | 0.0105 (±0.0032) |
| Hydrogenedentes | 1.029 | *P* = 0.414 | 0.0094 (±0.0022) | 0.0075 (±0.0026) | 0.0179 (±0.0061) | 0.0073 (±0.0044) | 0.0112 (±0.0018) | 0.0119 (±0.0029) |
| Tenericutes | 1.181 | *P* = 0.336 | 0.0084 (±0.0031) | 0.0056 (±0.0032) | 0.0311 (±0.0129) | 0.0031 (±0.0016) | 0.0212 (±0.0069) | 0.1052 (±0.0928) |
| Spirochaetes | 1.434 | *P* = 0.234 | 0.0071 (±0.0020) | 0.0027 (±0.0027) | 0.0010 (±0.0006) | 0.0050 (±0.0025) | 0.0038 (±0.0011) | 0.0031 (±0.0011) |
| Halanaerobiaeota | 1.025 | *P* = 0.416 | 0.0047 (±0.0015) | 0.0037 (±0.0015) | 0.0082 (±0.0021) | 0.0022 (±0.0022) | 0.0064 (±0.0011) | 0.0050 (±0.0017) |
| WS2 | 1.744 | *P* = 0.148 | 0.0034 (±0.0014) | 0.0125 (±0.0031) | 0.0053 (±0.0008) | 0.0086 (±0.0027) | 0.0068 (±0.0011) | 0.0095 (±0.0041) |
| GAL15 | 2.884 | *P* = 0.026 | 0.0023 (±0.0015) | n.d | 0.0014 (±0.0010) ^ns^ | 0.0039 (±0.0020) ^ns^ | 0.0004 (±0.0003) ^ns^ | 0.0040 (±0.0016) ^ns^ |
| Zixibacteria | 0.187 | *P* = 0.966 | 0.0022 (±0.0015) | 0.0019 (±0.0019) | 0.0027 (±0.0010) | 0.0039 (±0.0024) | 0.0020 (±0.0008) | 0.0024 (±0.0008) |
| WPS-2 | 2.045 | *P* = 0.094 | 0.0019 (±0.0012) | 0.0010 (±0.0010) | 0.0074 (±0.0027) | n.d | 0.0057 (±0.0017) | n.d |
| Deinococcus_Thermus | 4.406 | *P* = 0.003 | 0.0007 (±0.0007) | n.d | 0.0021 (±0.0011) ^ns^ | 0.0031 (±0.0016)* | n.d | n.d |
| Margulisbacteria | 0.373 | *P* = 0.864 | 0.0005 (±0.0005) | n.d | n.d | n.d | 0.0004 (±0.0003) | 0.0005 (±0.0005) |
| Calditrichaeota | 0.582 | *P* = 0.713 | n.d | n.d | 0.0005 (±0.0005) | n.d | 0.0009 (±0.0005) | n.d |
| Dadabacteria | 4.413 | *P* = 0.003 | n.d | 0.0047 (±0.0029) | n.d | 0.0017 (±0.0017) | 0.0003 (±0.0003) | n.d |
| FBP | 0.372 | *P* = 0.864 | n.d | n.d | 0.0005 (±0.0005) | n.d | 0.0004 (±0.0003) | 0.0006 (±0.0006) |

Table S3: The effect of sward type on the relative abundance (%) of the classified genera within the fungal phylum *Glomeromycota* at the end of the three year conditioning phase (March). The effect of sward type and their differences were tested with ANOVA. Corresponding *F* – and *P* – values refer to the overall effect, and the significance tests to sward types are against Lp, based on post-hoc contrasts. Genera are ordered by % relative abundance in the Lp monoculture (highest to lowest). Abbreviations are explained in the legend of Figure 1 in the main text. Significance codes: ‘***’ *P* **≤** 0.001 ‘**’ *P* **≤** 0.01 ‘*’ *P* **≤** 0.05 ‘ns*’ P >* 0.05. ‘n.d’ signifies no detection.

|  |  |  |  |  |  |  | Sward type |  |  |  |  |  |  |
| --- | --- | --- | --- | --- | --- | --- | --- | --- | --- | --- | --- | --- | --- |
|  | *F* - value | *P* - value | Lp |  | Ci |  | Tp |  | Lp–Ci |  | Lp–Tp |  | Lp–Ci–Tp |
| *Claroideoglomus* | 7.214 | *P* < 0.001 | 0.209 (±0.048) |  | 0.237 (±0.072)^ns^ |  | 0.050 (±0.014)*** |  | 0.143 (±0.030)^ns^ |  | 0.068 (±0.012)*** |  | 0.118 (±0.010)* |
| *Paraglomus* | 2.139 | *P* = 0.081 | 0.200 (±0.031) |  | 0.121 (±0.041) |  | 0.004 (±0.001) |  | 0.176 (±0.040) |  | 0.112 (±0.036) |  | 0.082 (±0.035) |
| *Glomus* | 8.315 | *P* < 0.001 | 0.184 (±0.045) |  | 0.252 (±0.102)^ns^ |  | 0.012 (±0.004)*** |  | 0.158 (±0.039)^ns^ |  | 0.045 (±0.009)*** |  | 0.091 (±0.020)* |
| *Funneliformis* | 5.169 | *P* < 0.001 | 0.059 (±0.012) |  | 0.086 (±0.005)^ns^ |  | 0.029 (±0.012)^ns^ |  | 0.025 (±0.002)^ns^ |  | 0.053 (±0.007)^ns^ |  | 0.100 (±0.014)* |
| *Dominikia* | 7.498 | *P* < 0.001 | 0.059 (±0.010) |  | 0.052 (±0.020)^ns^ |  | 0.007 (±0.003)*** |  | 0.043 (±0.030)^ns^ |  | 0.009 (±0.003)*** |  | 0.025 (±0.009)* |
| *Archaeospora* | 2.811 | *P* = 0.029 | 0.028 (±0.008) |  | 0.006 (±0.004)* |  | 0.0004 (±0.0004)** |  | 0.003 (±0.002)* |  | 0.013 (±0.004)* |  | 0.004 (±0.002)** |
| *Rhizophagus* | 2.237 | *P* = 0.070 | 0.025 (±0.009) |  | 0.019 (±0.004) |  | 0.006 (±0.003) |  | 0.020 (±0.011) |  | 0.009 (±0.002) |  | 0.011 (±0.005) |
| *Septoglomus* | 8.006 | *P* < 0.001 | 0.020 (±0.004) |  | 0.031 (±0.014) ^ns^ |  | n.d |  | 0.018 (±0.013) ^ns^ |  | 0.002 (±0.001)*** |  | 0.011 (±0.004) ^ns^ |
| *Cetraspora* | 1.223 | *P* = 0.317 | 0.009 (±0.005) |  | 0.001 (±0.001) |  | 0.008 (±0.004) |  | 0.023 (±0.021) |  | 0.005 (±0.002) |  | 0.010 (±0.007) |
| *Ambispora* | 2.024 | *P* = 0.097 | 0.009 (±0.006) |  | 0.001 (±0.001) |  | n.d |  | n.d |  | 0.001 (±0.001) |  | 0.002 (±0.002) |
| *Diversispora* | 10.19 | *P* < 0.001 | 0.008 (±0.002) |  | 0.008 (±0.003) |  | n.d |  | 0.010 (±0.003) |  | 0.001 (±0.0005)*** |  | 0.002 (±0.001)** |
| *Scutellospora* | 3.879 | *P* < 0.001 | 0.002 (±0.001) |  | 0.016 (±0.008)** |  | 0.003 (±0.002)^ns^ |  | n.d |  | 0.002 (±0.001)^ns^ |  | 0.008 (±0.004)^ns^ |
| *Gigaspora* | 1.057 | *P* = 0.399 | 0.002 (±0.002) |  | n.d |  | n.d |  | n.d |  | 0.0002 (±0.0002) |  | n.d |
| *Acaulospora* | 2.619 | *P* = 0.039 | n.d |  | n.d |  | n.d |  | 0.007 (±0.007) |  | n.d |  | 0.002 (±0.002) |

Table S4: Fungal OTUs (fOTUs), which were detected as unique indicator OTUs for each monoculture sward at the end of the three year conditioning phase in March, through indicator species analysis (IndVal > 0.8, *P* ≤ 0.05). The taxonomy of each detected OTU is given at the lowest known taxonomic level (down to the genus level):- k_: kingdom, p_: phylum, c_: class, o_: order, f_: family, g_: genus. Given is the mean relative abundance (%, ± 1 SE) for each OTU (ab.) as well as the % change in abundance between the indicator monoculture and the two non-indicator monocultures. Also shown is whether the indicator OTU detected in March was also detected as such during the legacy samplings (June, August and September, when the monocultures were removed and replaced with a *Lolium multiflorum* following crop), with ✔ indicating it was and X indicating it was not. Abbreviations are explained in the legend of Figure 1.

| OTU | Taxonomy | IndVal | *P* - value | Plant | ab. | % Change | | legacy | | |
| --- | --- | --- | --- | --- | --- | --- | --- | --- | --- | --- |
| Fungi |  |  |  |  |  | Ci | Tp | June | August | September |
| fOTU_1145 | g__Ramularia | 1 | *P* < 0.001 | Lp | 0.009 | -96.17 | -85.74 | X | X | X |
| fOTU_1406 | p__Basidiomycota | 1 | *P* < 0.001 | Lp | 0.007 | -79 | -89.89 | X | X | X |
| fOTU_2217 | g__Clavatospora | 1 | *P* < 0.001 | Lp | 0.002 | n.d | n.d | X | X | X |
| fOTU_167 | c__Sordariomycetes | 0.999 | *P* = 0.001 | Lp | 0.309 | -93.18 | -99.58 | ✔ | ✔ | X |
| fOTU_759 | g__Phaeosphaeria | 0.998 | *P* = 0.001 | Lp | 0.032 | n.d | -98.7 | X | X | X |
| fOTU_223 | g__Phaeosphaeria | 0.998 | *P* < 0.001 | Lp | 0.144 | -78.66 | -90.58 | ✔ | X | X |
| fOTU_412 | g__unclassified_Tubeufiaceae | 0.995 | *P* < 0.001 | Lp | 0.063 | -93.63 | -98.59 | ✔ | ✔ | X |
| fOTU_270 | p__Ascomycota | 0.995 | *P* = 0.001 | Lp | 0.149 | -95.16 | -99.39 | ✔ | ✔ | ✔ |
| fOTU_35 | p__Basidiomycota | 0.994 | *P* = 0.030 | Lp | 1.877 | -49.54 | -78.68 | X | X | X |
| fOTU_918 | o__Hypocreales | 0.989 | *P* < 0.001 | Lp | 0.025 | -96.83 | -95.55 | ✔ | ✔ | ✔ |
| fOTU_4737 | g__Volucrispora | 0.988 | *P* < 0.001 | Lp | 0.055 | -95.17 | -97.55 | X | X | X |
| fOTU_603 | c__Sordariomycetes | 0.988 | *P* < 0.001 | Lp | 0.046 | -92.36 | -97.76 | ✔ | X | X |
| fOTU_353 | g__Pyrenochaeta | 0.984 | *P* = 0.001 | Lp | 0.102 | -97.64 | -97.83 | ✔ | ✔ | X |
| fOTU_1046 | g__Serendipita | 0.981 | *P* < 0.001 | Lp | 0.063 | -97.41 | -93.54 | ✔ | ✔ | X |
| fOTU_400 | c__Sordariomycetes | 0.980 | *P* < 0.001 | Lp | 0.048 | -77.43 | -94.61 | ✔ | X | X |
| fOTU_4500 | g__Articulospora | 0.975 | *P* = 0.002 | Lp | 0.149 | -97.06 | -90.69 | ✔ | X | X |
| fOTU_859 | g__unclassified_Microbotryomycetes | 0.974 | *P* < 0.001 | Lp | 0.024 | -89.33 | -97.53 | ✔ | ✔ | ✔ |
| fOTU_499 | p__Ascomycota | 0.974 | *P* < 0.001 | Lp | 0.014 | -96.37 | -76.17 | X | X | X |
| fOTU_2793 | f__Lasiosphaeriaceae | 0.972 | *P* = 0.002 | Lp | 0.131 | -72.84 | -97.64 | X | ✔ | X |
| fOTU_565 | g__Schizothecium | 0.968 | *P* = 0.001 | Lp | 0.059 | -61.01 | -90.04 | X | X | X |
| fOTU_220 | g__unclassified_Ceratobasidiaceae | 0.968 | *P* = 0.001 | Lp | 0.106 | -77.06 | +22.1 | X | X | X |
| fOTU_299 | g__Paraglomus | 0.965 | *P* = 0.001 | Lp | 0.057 | -85.89 | -66.18 | X | X | X |
| fOTU_80 | g__Slooffia | 0.963 | *P* < 0.001 | Lp | 0.665 | -93.68 | -93.18 | ✔ | ✔ | X |
| fOTU_163 | o__Auriculariales | 0.962 | *P* < 0.001 | Lp | 0.198 | -69.13 | -96.29 | X | X | X |
| fOTU_133 | f__Glomerellaceae | 0.960 | *P* < 0.001 | Lp | 0.122 | +119.12 | -53.33 | ✔ | X | X |
| fOTU_3006 | f__Didymellaceae | 0.960 | *P* = 0.001 | Lp | 0.024 | +155.62 | -8.14 | X | X | X |
| fOTU_302 | o__Pleosporales | 0.958 | *P* < 0.001 | Lp | 0.095 | -89.61 | -96.85 | X | ✔ | X |
| fOTU_204 | c__Sordariomycetes | 0.957 | *P* = 0.001 | Lp | 0.109 | -88.08 | -60.26 | X | X | X |

Table S4: continued

| OTU | Taxonomy | IndVal | *P* - value | Plant | ab. | % Change | | legacy | | |
| --- | --- | --- | --- | --- | --- | --- | --- | --- | --- | --- |
| Fungi |  |  |  |  |  | Ci | Tp | June | August | September |
| fOTU_3406 | f__Lasiosphaeriaceae | 0.956 | *P* = 0.003 | Lp | 0.116 | -47.16 | -98.73 | X | X | X |
| fOTU_1658 | k__Fungi | 0.954 | *P* = 0.001 | Lp | 0.017 | -90.15 | -98.97 | ✔ | ✔ | ✔ |
| fOTU_101 | o__Pleosporales | 0.951 | *P* = 0.002 | Lp | 0.059 | -30.35 | +229.62 | X | X | X |
| fOTU_155 | c__Sordariomycetes | 0.948 | *P* = 0.003 | Lp | 0.278 | -94.68 | -95.4 | X | X | ✔ |
| fOTU_1040 | g__Dioszegia | 0.946 | *P* = 0.001 | Lp | 0.010 | n.d | -88.16 | X | X | X |
| fOTU_209 | g__Phomatospora | 0.944 | *P* < 0.001 | Lp | 0.159 | -71.58 | -89.67 | ✔ | X | X |
| fOTU_175 | o__Pleosporales | 0.938 | *P* = 0.03 | Lp | 0.170 | -80.03 | -74.41 | X | X | X |
| fOTU_3698 | g__Glarea | 0.928 | *P* = 0.012 | Lp | 0.166 | -93.53 | -87.48 | ✔ | X | X |
| fOTU_445 | g__unclassified_Stachybotryaceae | 0.916 | *P* = 0.017 | Lp | 0.067 | -90.7 | -96.11 | X | X | ✔ |
| fOTU_1061 | k__Fungi | 0.913 | *P* = 0.003 | Lp | 0.059 | -89.77 | n.d | ✔ | ✔ | ✔ |
| fOTU_1677 | g__Cyphellophora | 0.913 | *P* = 0.003 | Lp | 0.009 | -88.77 | n.d | X | X | X |
| fOTU_1760 | g__Archaeospora | 0.913 | *P* = 0.002 | Lp | 0.003 | n.d | -80.64 | X | X | X |
| fOTU_197 | g__Microscypha | 0.913 | *P* = 0.003 | Lp | 0.172 | -85.3 | -96.32 | X | ✔ | X |
| fOTU_2620 | p__Basidiomycota | 0.913 | *P* = 0.003 | Lp | 0.003 | n.d | n.d | X | X | X |
| fOTU_3002 | f__Kondoaceae | 0.913 | *P* = 0.003 | Lp | 0.003 | n.d | n.d | X | X | X |
| fOTU_354 | o__Helotiales | 0.913 | *P* = 0.003 | Lp | 0.022 | +281.48 | -93.25 | X | X | X |
| fOTU_525 | g__Coprinopsis | 0.913 | *P* = 0.003 | Lp | 0.084 | -97.36 | n.d | ✔ | ✔ | ✔ |
| fOTU_660 | c__Sordariomycetes | 0.913 | *P* = 0.003 | Lp | 0.084 | -96.71 | -99.35 | ✔ | ✔ | ✔ |
| fOTU_121 | g__unclassified_Phaeosphaeriaceae | 0.912 | *P* = 0.004 | Lp | 0.432 | -92.71 | -87.99 | ✔ | X | X |
| fOTU_180 | p__Ascomycota | 0.900 | *P* = 0.004 | Lp | 0.135 | -72.4 | -49.01 | X | X | X |
| fOTU_784 | g__Ophiosphaerella | 0.898 | *P* = 0.006 | Lp | 0.035 | -94.53 | -97.24 | X | ✔ | X |
| fOTU_1175 | f__Cystobasidiaceae | 0.898 | *P* = 0.004 | Lp | 0.005 | -72.29 | -72.9 | X | X | X |
| fOTU_4565 | g__unclassified_Glomeraceae | 0.897 | *P* = 0.004 | Lp | 0.009 | -58.22 | -80.96 | X | X | X |
| fOTU_1802 | c__Microbotryomycetes | 0.877 | *P* = 0.006 | Lp | 0.003 | n.d | -70.04 | X | X | X |
| fOTU_2101 | g__unclassified_Glomeraceae | 0.877 | *P* = 0.011 | Lp | 0.027 | -26.79 | -84.74 | X | X | X |
| fOTU_195 | g__Alternaria | 0.874 | *P* = 0.014 | Lp | 0.103 | -57.64 | -22.43 | X | X | X |
| fOTU_1518 | k__Fungi | 0.872 | *P* = 0.013 | Lp | 0.013 | n.d | -90.98 | X | X | X |
| fOTU_1667 | f__Glomeraceae | 0.861 | *P* = 0.008 | Lp | 0.005 | -67.68 | -71.63 | X | ✔ | X |

Table S4: continued

| OTU | Taxonomy | IndVal | *P* - value | Plant | ab. | % Change | | legacy | | |
| --- | --- | --- | --- | --- | --- | --- | --- | --- | --- | --- |
| Fungi |  |  |  |  |  | Ci | Tp | June | August | September |
| fOTU_583 | g__unclassified_Agaricales | 0.847 | *P* = 0.006 | Lp | 0.099 | -98.02 | -92.27 | X | X | X |
| fOTU_3724 | g__unclassified_Tubeufiaceae | 0.845 | *P* = 0.016 | Lp | 0.012 | +54.83 | -99.03 | X | ✔ | X |
| fOTU_1449 | g__unclassified_Sebacinales | 0.845 | *P* = 0.022 | Lp | 0.009 | -6.69 | -98.71 | X | X | X |
| fOTU_4884 | g__Talaromyces | 0.844 | *P* = 0.008 | Lp | 0.005 | -74.42 | -80.42 | X | X | X |
| fOTU_848 | o__Hypocreales | 0.844 | *P* = 0.008 | Lp | 0.009 | -94.85 | -87.18 | ✔ | ✔ | X |
| fOTU_1048 | p__Chytridiomycota | 0.838 | *P* = 0.022 | Lp | 0.009 | n.d | -70.19 | X | X | X |
| fOTU_113 | o__Helotiales | 0.826 | *P* = 0.009 | Lp | 0.220 | +8.78 | -93.92 | ✔ | X | X |
| fOTU_1017 | k__Fungi | 0.816 | *P* = 0.019 | Lp | 0.007 | -92.13 | -96.95 | X | X | X |
| fOTU_1079 | o__Pleosporales | 0.816 | *P* = 0.023 | Lp | 0.008 | -97.47 | -91.51 | X | X | X |
| fOTU_1235 | k__Fungi | 0.816 | *P* = 0.019 | Lp | 0.006 | n.d | -98.55 | X | X | X |
| fOTU_1938 | g__unclassified_Corticiaceae | 0.816 | *P* = 0.016 | Lp | 0.004 | -56.48 | -68.04 | X | X | X |
| fOTU_2023 | g__Rhizophagus | 0.816 | *P* = 0.024 | Lp | 0.011 | n.d | n.d | ✔ | ✔ | ✔ |
| fOTU_215 | f__Didymosphaeriaceae | 0.816 | *P* = 0.012 | Lp | 0.156 | -79.7 | -86.77 | X | X | X |
| fOTU_2716 | g__Bensingtonia | 0.816 | *P* = 0.020 | Lp | 0.001 | n.d | n.d | X | X | X |
| fOTU_3397 | k__Fungi | 0.816 | *P* = 0.017 | Lp | 0.002 | n.d | n.d | X | X | X |
| fOTU_3403 | k__Fungi | 0.816 | *P* = 0.018 | Lp | 0.002 | n.d | n.d | X | X | X |
| fOTU_3697 | g__Septoglomus | 0.816 | *P* = 0.013 | Lp | 0.001 | -83.95 | -85.76 | X | X | X |
| fOTU_392 | o__Pleosporales | 0.816 | *P* = 0.011 | Lp | 0.012 | -65.59 | -83.42 | X | X | X |
| fOTU_4149 | k__Fungi | 0.816 | *P* = 0.012 | Lp | 0.001 | -83.95 | n.d | X | X | X |
| fOTU_4344 | k__Fungi | 0.816 | *P* = 0.022 | Lp | 0.001 | -63.72 | -20.22 | X | X | X |
| fOTU_576 | g__Calyptella | 0.816 | *P* = 0.013 | Lp | 0.020 | +35.1 | n.d | ✔ | X | X |
| fOTU_953 | g__unclassified_Tubeufiaceae | 0.816 | *P* = 0.018 | Lp | 0.033 | -83.01 | n.d | X | ✔ | X |
| fOTU_485 | g__unclassified_Chaetothyriaceae | 0.807 | *P* = 0.033 | Lp | 0.071 | -94.75 | -98.66 | X | ✔ | ✔ |
| fOTU_2152 | k__Fungi | 0.803 | *P* = 0.019 | Lp | 0.010 | -88.35 | -98.87 | X | ✔ | X |
| fOTU_2532 | f__Kondoaceae | 0.802 | *P* = 0.023 | Lp | 0.002 | n.d | -72.46 | X | X | X |
|  |  |  |  |  |  | Lp | Leg |  |  |  |
| fOTU_1301 | k__Fungi | 1 | *P* = 0.001 | Ci | 0.024 | n.d | -98.18 | X | X | X |
| fOTU_806 | g__Tricharina | 1 | *P* = 0.001 | Ci | 0.016 | n.d | n.d | X | X | X |

Table S4: continued

| OTU | Taxonomy | IndVal | *P* - value | Plant | ab. | % Change | | legacy | | |
| --- | --- | --- | --- | --- | --- | --- | --- | --- | --- | --- |
| Fungi |  |  |  |  |  | Ci | Tp | June | August | September |
| fOTU_185 | c__Eurotiomycetes | 1 | *P* < 0.001 | Ci | 0.363 | -78.14 | -99.87 | ✔ | ✔ | X |
| fOTU_1068 | k__Fungi | 0.984 | *P* < 0.001 | Ci | 0.019 | -98.17 | -96.01 | ✔ | X | X |
| fOTU_236 | g__Tetracladium | 0.984 | *P* = 0.003 | Ci | 0.296 | -98.79 | -96.39 | ✔ | ✔ | X |
| fOTU_910 | k__Fungi | 0.981 | *P* = 0.001 | Ci | 0.059 | -94.44 | -99.81 | ✔ | ✔ | ✔ |
| fOTU_963 | g__Itersonilia | 0.972 | *P* = 0.002 | Ci | 0.036 | -97.55 | -96.11 | X | X | X |
| fOTU_1643 | g__Protrudomyces | 0.956 | *P* < 0.001 | Ci | 0.005 | n.d | -91.59 | X | X | X |
| fOTU_3956 | g__Stagonosporopsis | 0.955 | *P* = 0.001 | Ci | 0.124 | -99.17 | -97.92 | ✔ | ✔ | ✔ |
| fOTU_588 | c__Sordariomycetes | 0.950 | *P* = 0.017 | Ci | 0.074 | -94.93 | -94.15 | X | ✔ | X |
| fOTU_553 | g__Hyalorbilia | 0.944 | *P* = 0.011 | Ci | 0.165 | -83.76 | -99.37 | X | X | X |
| fOTU_600 | g__Talaromyces | 0.923 | *P* = 0.013 | Ci | 0.036 | -64.69 | -89.96 | X | X | X |
| fOTU_2632 | k__Fungi | 0.921 | *P* = 0.002 | Ci | 0.002 | -92.61 | -80.56 | X | X | X |
| fOTU_145 | g__Chaetosphaeronema | 0.921 | *P* = 0.011 | Ci | 0.402 | -98.6 | -78.56 | ✔ | X | X |
| fOTU_762 | g__Colletotrichum | 0.919 | *P* = 0.002 | Ci | 0.022 | -70.86 | -71.18 | X | X | X |
| fOTU_5528 | g__Cladosporium | 0.918 | *P* = 0.003 | Ci | 0.010 | -91.61 | -96.39 | X | X | X |
| fOTU_770 | g__Clavatospora | 0.918 | *P* = 0.002 | Ci | 0.011 | -93.44 | -83.41 | X | X | ✔ |
| fOTU_519 | o__Pezizales | 0.915 | *P* = 0.004 | Ci | 0.045 | -80.56 | -98.39 | ✔ | X | X |
| fOTU_1099 | k__Fungi | 0.907 | *P* = 0.002 | Ci | 0.009 | -92.47 | -86.94 | ✔ | X | X |
| fOTU_1426 | k__Fungi | 0.894 | *P* = 0.009 | Ci | 0.007 | -80.8 | -69.52 | X | X | X |
| fOTU_3885 | g__Lipomyces | 0.889 | *P* = 0.005 | Ci | 0.004 | -55.63 | -93.07 | X | X | X |
| fOTU_631 | o__Pleosporales | 0.880 | *P* = 0.010 | Ci | 0.053 | -86.45 | -93.74 | ✔ | ✔ | X |
| fOTU_1871 | p__Basidiomycota | 0.876 | *P* = 0.006 | Ci | 0.003 | -86.97 | -75.07 | X | X | X |
| fOTU_1121 | p__Chytridiomycota | 0.866 | *P* = 0.007 | Ci | 0.008 | -52.93 | -69.49 | X | X | X |
| fOTU_1342 | g__Clavaria | 0.866 | *P* = 0.005 | Ci | 0.035 | -89.92 | n.d | X | X | X |
| fOTU_1379 | g__Coralloidiomyces | 0.866 | *P* = 0.007 | Ci | 0.032 | -99.69 | n.d | X | X | X |
| fOTU_1589 | p__Ascomycota | 0.866 | *P* = 0.007 | Ci | 0.011 | n.d | n.d | X | X | X |
| fOTU_2059 | k__Fungi | 0.866 | *P* = 0.007 | Ci | 0.007 | -91.87 | -95.77 | X | ✔ | X |
| fOTU_2600 | o__Rhizophydiales | 0.866 | *P* = 0.008 | Ci | 0.002 | -76.32 | -47.44 | X | X | X |
| fOTU_2708 | g__Alternaria | 0.866 | *P* = 0.008 | Ci | 0.003 | n.d | n.d | X | X | X |

Table S4: continued

| OTU | Taxonomy | IndVal | *P* - value | Plant | ab. | % Change | | legacy | | |
| --- | --- | --- | --- | --- | --- | --- | --- | --- | --- | --- |
| Fungi |  |  |  |  |  | Ci | Tp | June | August | September |
| fOTU_3081 | k__Fungi | 0.866 | *P* = 0.007 | Ci | 0.002 | n.d | n.d | X | X | X |
| fOTU_3092 | k__Fungi | 0.866 | *P* = 0.007 | Ci | 0.004 | n.d | n.d | X | X | X |
| fOTU_3560 | k__Fungi | 0.866 | *P* = 0.008 | Ci | 0.002 | -70.89 | n.d | X | X | X |
| fOTU_672 | f__Ceratobasidiaceae | 0.864 | *P* = 0.004 | Ci | 0.040 | n.d | -93.95 | X | X | X |
| fOTU_2938 | g__Penicillium | 0.861 | *P* = 0.034 | Ci | 0.349 | -97.05 | -80.99 | ✔ | X | X |
| fOTU_440 | o__Pleosporales | 0.860 | *P* = 0.009 | Ci | 0.115 | -99.91 | -97.55 | ✔ | X | X |
| fOTU_1825 | k__Fungi | 0.857 | *P* = 0.015 | Ci | 0.008 | n.d | -98.06 | X | X | X |
| fOTU_3048 | k__Fungi | 0.857 | *P* = 0.007 | Ci | 0.004 | -79.65 | -92.04 | X | X | X |
| fOTU_4706 | c__Agaricomycetes | 0.850 | *P* = 0.005 | Ci | 0.005 | -89.37 | n.d | X | X | X |
| fOTU_2151 | o__Rhizophydiales | 0.850 | *P* = 0.007 | Ci | 0.004 | -96.68 | n.d | X | X | X |
| fOTU_1817 | g__unclassified_Entylomatales | 0.849 | *P* = 0.004 | Ci | 0.004 | -56.59 | -94.39 | X | X | X |
| fOTU_1134 | c__Agaricomycetes | 0.848 | *P* = 0.007 | Ci | 0.004 | -68.29 | n.d | X | ✔ | X |
| fOTU_1149 | c__Eurotiomycetes | 0.847 | *P* = 0.009 | Ci | 0.021 | -60.74 | n.d | X | X | X |
| fOTU_242 | g__Acremonium | 0.846 | *P* = 0.004 | Ci | 0.094 | -90.41 | -87.48 | X | X | X |
| fOTU_962 | f__Spizellomycetaceae | 0.846 | *P* = 0.005 | Ci | 0.009 | -29.47 | -81.38 | X | X | X |
| fOTU_1357 | g__Lophiotrema | 0.845 | *P* = 0.003 | Ci | 0.008 | n.d | -95.37 | ✔ | ✔ | X |
| fOTU_2162 | k__Fungi | 0.843 | *P* = 0.008 | Ci | 0.005 | -93.85 | n.d | X | X | X |
| fOTU_572 | g__Clavaria | 0.841 | *P* = 0.007 | Ci | 0.062 | -97.31 | -99.43 | ✔ | X | X |
| fOTU_2191 | g__Funneliformis | 0.839 | *P* = 0.011 | Ci | 0.015 | -93.99 | -86.52 | X | ✔ | X |
| fOTU_3094 | k__Fungi | 0.830 | *P* = 0.007 | Ci | 0.001 | -81.96 | -69.41 | X | X | X |
| fOTU_1580 | g__Wallemia | 0.829 | *P* = 0.028 | Ci | 0.023 | -94.7 | -94.44 | ✔ | X | X |
| fOTU_1186 | k__Fungi | 0.827 | *P* = 0.010 | Ci | 0.008 | -69.09 | -89.95 | X | X | X |
| fOTU_1470 | g__Clavatospora | 0.819 | *P* = 0.012 | Ci | 0.006 | -81.69 | -73.96 | X | X | X |
| fOTU_2535 | o__Glomerales | 0.818 | *P* = 0.046 | Ci | 0.004 | -70.42 | -85.69 | X | X | X |
| fOTU_4452 | g__Vishniacozyma | 0.816 | *P* = 0.017 | Ci | 0.004 | -83.91 | -36.89 | X | X | X |
|  |  |  |  |  |  | Lp | Ci |  |  |  |
| fOTU_256 | o__Xylariales | 1 | *P* < 0.001 | Tp | 0.131 | -99.25 | -99.67 | ✔ | ✔ | ✔ |
| fOTU_293 | k__Fungi | 0.989 | *P* < 0.001 | Tp | 0.141 | -99.5 | -95.75 | ✔ | ✔ | ✔ |

Table S4: continued

| OTU | Taxonomy | IndVal | *P* - value | Plant | ab. | % Change | | legacy | | |
| --- | --- | --- | --- | --- | --- | --- | --- | --- | --- | --- |
| Fungi |  |  |  |  |  | Lp | Ci | June | August | September |
| fOTU_4850 | g__Plectosphaerella | 0.987 | *P* < 0.001 | Tp | 0.012 | -95.54 | n.d | ✔ | ✔ | ✔ |
| fOTU_483 | o__Hypocreales | 0.986 | *P* < 0.001 | Tp | 0.021 | -97.78 | -97.16 | ✔ | ✔ | X |
| fOTU_179 | o__Helotiales | 0.984 | *P* < 0.001 | Tp | 0.140 | -97.15 | -95 | ✔ | ✔ | X |
| fOTU_332 | o__Helotiales | 0.981 | *P* = 0.001 | Tp | 0.118 | -97.69 | -98 | ✔ | ✔ | ✔ |
| fOTU_4968 | o__Helotiales | 0.980 | *P* < 0.001 | Tp | 0.031 | -95.07 | -98.33 | ✔ | X | X |
| fOTU_268 | o__Pleosporales | 0.979 | *P* = 0.001 | Tp | 0.139 | -98.31 | -97.78 | ✔ | ✔ | ✔ |
| fOTU_341 | o__Hypocreales | 0.978 | *P* < 0.001 | Tp | 0.066 | n.d | -94.18 | ✔ | ✔ | ✔ |
| fOTU_623 | g__unclassified_Branch06 | 0.976 | *P* < 0.001 | Tp | 0.026 | -98.35 | -97.15 | X | ✔ | ✔ |
| fOTU_301 | o__Hypocreales | 0.975 | *P* = 0.001 | Tp | 0.066 | -92.55 | -90.59 | X | ✔ | ✔ |
| fOTU_207 | o__Hypocreales | 0.952 | *P* = 0.001 | Tp | 0.112 | -94.31 | -90.83 | X | X | X |
| fOTU_253 | g__Leptosphaeria | 0.939 | *P* = 0.005 | Tp | 0.093 | -86.91 | -92.37 | ✔ | X | ✔ |
| fOTU_534 | g__Triscelophorus | 0.929 | *P* = 0.001 | Tp | 0.016 | -95.12 | -77.36 | ✔ | X | X |
| fOTU_329 | o__Helotiales | 0.926 | *P* = 0.003 | Tp | 0.035 | -47.02 | -93.64 | X | X | X |
| fOTU_527 | c__Dothideomycetes | 0.926 | *P* = 0.003 | Tp | 0.024 | -98.69 | n.d | X | X | X |
| fOTU_573 | g__Ascobolus | 0.926 | *P* = 0.003 | Tp | 0.022 | n.d | -66.75 | ✔ | X | X |
| fOTU_633 | g__unclassified_Ascomycota | 0.926 | *P* = 0.001 | Tp | 0.019 | n.d | n.d | ✔ | X | X |
| fOTU_274 | o__Pleosporales | 0.924 | *P* = 0.003 | Tp | 0.069 | -99.21 | -98.65 | ✔ | X | X |
| fOTU_159 | k__Fungi | 0.920 | *P* = 0.006 | Tp | 0.407 | -97.82 | -99.7 | ✔ | ✔ | ✔ |
| fOTU_230 | k__Fungi | 0.918 | *P* = 0.011 | Tp | 0.079 | -96.11 | -57.8 | ✔ | ✔ | X |
| fOTU_831 | p__Ascomycota | 0.895 | *P* = 0.007 | Tp | 0.019 | n.d | -91.63 | X | X | X |
| fOTU_187 | g__Conlarium | 0.893 | *P* = 0.010 | Tp | 0.143 | -85.49 | -98.84 | X | X | X |
| fOTU_1455 | k__Fungi | 0.890 | *P* = 0.006 | Tp | 0.019 | n.d | -91.22 | X | X | X |
| fOTU_925 | p__Ascomycota | 0.886 | *P* = 0.015 | Tp | 0.013 | -97.51 | -74.92 | X | X | X |
| fOTU_222 | g__Cercophora | 0.857 | *P* = 0.027 | Tp | 0.089 | -97.47 | +2.3 | X | ✔ | X |
| fOTU_1009 | o__Helotiales | 0.845 | *P* = 0.006 | Tp | 0.010 | -96.61 | n.d | X | X | X |
| fOTU_1225 | c__Agaricomycetes | 0.845 | *P* = 0.008 | Tp | 0.007 | n.d | n.d | X | X | X |
| fOTU_1439 | k__Fungi | 0.845 | *P* = 0.007 | Tp | 0.013 | -87.84 | -93.57 | X | ✔ | X |
| fOTU_1533 | k__Fungi | 0.845 | *P* = 0.007 | Tp | 0.004 | -66.79 | -55.99 | X | X | X |

Table S4: continued

| OTU | Taxonomy | IndVal | *P* - value | Plant | ab. | % Change | | legacy | | |
| --- | --- | --- | --- | --- | --- | --- | --- | --- | --- | --- |
| Fungi |  |  |  |  |  | Lp | Ci | June | August | September |
| fOTU_779 | g__unclassified_Halosphaeriaceae | 0.845 | *P* = 0.006 | Tp | 0.007 | -98.53 | n.d | ✔ | X | X |
| fOTU_915 | g__unclassified_Archaeosporales | 0.845 | *P* = 0.006 | Tp | 0.033 | -98.47 | n.d | X | ✔ | ✔ |
| fOTU_931 | o__Agaricales | 0.845 | *P* = 0.006 | Tp | 0.005 | n.d | -97.22 | ✔ | X | X |
| fOTU_747 | g__unclassified_Pucciniomycetes | 0.837 | *P* = 0.034 | Tp | 0.011 | -89.61 | -85.32 | X | X | X |
| fOTU_1254 | g__Beauveria | 0.833 | *P* = 0.030 | Tp | 0.005 | -91.41 | -94.27 | X | X | X |
| fOTU_641 | p__Ascomycota | 0.821 | *P* = 0.036 | Tp | 0.017 | -78.92 | -97.78 | X | X | X |
| fOTU_883 | f__Orbiliaceae | 0.810 | *P* = 0.024 | Tp | 0.009 | n.d | -67.89 | X | X | X |

Table S5: Bacterial OTUs (bOTUs), which were detected as unique indicator OTUs for each monoculture sward at the end of the three year conditioning phase in March, through indicator species analysis (IndVal > 0.8, *P* ≤ 0.05). The taxonomy of each detected OTU is given at the lowest known taxonomic level (down to the genus level):- k_: kingdom, p_: phylum, c_: class, o_: order, f_: family, g_: genus. Given is the mean relative abundance (%, ± 1 SE) for each OTU (ab.) as well as the % change in abundance between the indicator monoculture and the two non-indicator monocultures. Also shown is whether the indicator OTU detected in March was also detected as such during the legacy samplings (June, August and September, when the monocultures were removed and replaced with a *Lolium multiflorum* following crop), with ✔ indicating it was and X indicating it was not. Abbreviations are explained in the legend of Figure 1.

| OTU | Taxonomy | IndVal | *P* - value | Plant | ab. | % Change | | legacy | | |
| --- | --- | --- | --- | --- | --- | --- | --- | --- | --- | --- |
| Bacteria |  |  |  |  |  | Ci | Tp | June | August | September |
| bOTU_2706 | o_S-BQ2-57_soil_group | 0.971 | *P* < 0.001 | Lp | 0.006 | -87.8 | -85.16 | X | X | X |
| bOTU_9385 | g_Haliangium | 0.963 | *P* < 0.001 | Lp | 0.013 | -81.58 | -85.44 | ✔ | X | X |
| bOTU_1660 | f_CPla-3_termite_group | 0.956 | *P* = 0.001 | Lp | 0.013 | -92.24 | -85.96 | ✔ | X | X |
| bOTU_1009 | d_Bacteria | 0.953 | *P* < 0.001 | Lp | 0.051 | -63.05 | -89.45 | X | X | X |
| bOTU_4153 | g_BIyi10 | 0.930 | *P* < 0.001 | Lp | 0.003 | -58.35 | n.d | X | X | X |
| bOTU_1349 | g_Haliangium | 0.927 | *P* = 0.003 | Lp | 0.016 | -86.63 | -83.59 | ✔ | X | X |
| bOTU_6227 | f_BIrii41 | 0.924 | *P* = 0.002 | Lp | 0.013 | -79.98 | -88.22 | X | X | X |
| bOTU_11213 | o_Saccharimonadales | 0.913 | *P* = 0.003 | Lp | 0.001 | n.d | -71.54 | X | X | X |
| bOTU_1436 | f_CPla-3_termite_group | 0.913 | *P* = 0.002 | Lp | 0.011 | -89.69 | -68.51 | ✔ | X | X |
| bOTU_2306 | g_Steroidobacter | 0.913 | *P* = 0.003 | Lp | 0.004 | -69.27 | -46.07 | X | X | X |
| bOTU_2544 | f_A4b | 0.913 | *P* = 0.003 | Lp | 0.003 | -84.47 | -79.62 | X | X | X |
| bOTU_4682 | c_Parcubacteria | 0.913 | *P* = 0.002 | Lp | 0.002 | n.d | n.d | X | X | X |
| bOTU_6439 | o_SBR1031 | 0.913 | *P* = 0.002 | Lp | 0.003 | n.d | n.d | ✔ | X | ✔ |
| bOTU_2663 | p_Patescibacteria | 0.912 | *P* = 0.001 | Lp | 0.004 | -95.45 | -58.07 | X | X | X |
| bOTU_3498 | o_SBR1031 | 0.856 | *P* = 0.006 | Lp | 0.005 | n.d | -91.94 | X | ✔ | X |
| bOTU_1755 | o_Saccharimonadales | 0.853 | *P* = 0.007 | Lp | 0.007 | -90.03 | -33.31 | X | X | X |
| bOTU_6068 | c_Parcubacteria | 0.850 | *P* = 0.012 | Lp | 0.002 | n.d | -78.25 | X | X | X |
| bOTU_2925 | f_Gemmatimonadaceae | 0.850 | *P* = 0.006 | Lp | 0.004 | -71.43 | -51.4 | X | X | X |
| bOTU_2884 | d_Bacteria | 0.842 | *P* = 0.010 | Lp | 0.007 | -55.64 | -79.54 | X | X | X |
| bOTU_3926 | o_Candidatus_Magasanikbacteria | 0.837 | *P* = 0.010 | Lp | 0.003 | -79.31 | n.d | X | X | X |
| bOTU_2991 | f_Elsteraceae | 0.832 | *P* = 0.008 | Lp | 0.004 | -93.94 | -97.49 | X | ✔ | ✔ |
| bOTU_2624 | c_ABY1 | 0.828 | *P* = 0.020 | Lp | 0.003 | -41.43 | -57.62 | X | X | X |
| bOTU_2250 | d_Bacteria | 0.817 | *P* = 0.012 | Lp | 0.008 | -77.33 | -98.77 | X | ✔ | ✔ |
| bOTU_10205 | f_Caldilineaceae | 0.816 | *P* = 0.017 | Lp | 0.003 | -70.19 | -37.63 | X | X | X |
| bOTU_1579 | c_Subgroup_6 | 0.816 | *P* = 0.013 | Lp | 0.010 | -89.15 | -88.22 | ✔ | ✔ | ✔ |
| bOTU_2042 | o_Subgroup_7 | 0.816 | *P* = 0.014 | Lp | 0.003 | -27.92 | -82.61 | X | X | X |
| bOTU_2852 | f_Roseiflexaceae | 0.816 | *P* = 0.011 | Lp | 0.006 | -93.9 | -81.52 | ✔ | ✔ | X |
| bOTU_3060 | g_Nocardioides | 0.816 | *P* = 0.011 | Lp | 0.002 | -57.07 | -37.89 | X | X | X |

Table S5: continued

| OTU | Taxonomy | IndVal | *P* - value | Plant | ab. | % Change | | legacy | | |
| --- | --- | --- | --- | --- | --- | --- | --- | --- | --- | --- |
| Bacteria |  |  |  |  |  | Ci | Tp | June | August | September |
| bOTU_3883 | c_Subgroup_6 | 0.816 | *P* = 0.009 | Lp | 0.001 | -59.91 | -26.91 | X | X | X |
| bOTU_4135 | f_Gemmataceae | 0.816 | *P* = 0.007 | Lp | 0.001 | +5.44 | -58 | X | X | X |
| bOTU_4198 | g_Candidatus_Protochlamydia | 0.816 | *P* = 0.007 | Lp | 0.004 | -57.23 | -38.05 | X | X | X |
| bOTU_4747 | o_Candidatus_Kaiserbacteria | 0.816 | *P* = 0.019 | Lp | 0.004 | n.d | -96.39 | X | X | X |
| bOTU_4930 | f_Gemmataceae | 0.816 | *P* = 0.007 | Lp | 0.002 | n.d | -74.42 | X | X | X |
| bOTU_5061 | o_Saccharimonadales | 0.816 | *P* = 0.010 | Lp | 0.003 | -84.77 | -80.19 | X | X | X |
| bOTU_5239 | c_Gammaproteobacteria | 0.816 | *P* = 0.007 | Lp | 0.001 | -83.13 | -79.15 | X | X | X |
| bOTU_5258 | o_Saccharimonadales | 0.816 | *P* = 0.016 | Lp | 0.002 | n.d | n.d | X | X | X |
| bOTU_6140 | f_Chthoniobacteraceae | 0.816 | *P* = 0.011 | Lp | 0.001 | n.d | n.d | X | X | X |
| bOTU_6309 | g_Gemmata | 0.816 | *P* = 0.018 | Lp | 0.004 | -93.66 | -77.84 | X | X | X |
| bOTU_6542 | g_Kibdelosporangium | 0.816 | *P* = 0.007 | Lp | 0.002 | -85.33 | -89.59 | X | X | X |
| bOTU_7147 | c_Alphaproteobacteria | 0.816 | *P* = 0.011 | Lp | 0.004 | -93.74 | -92.52 | X | X | X |
| bOTU_7263 | f_BIrii41 | 0.816 | *P* = 0.017 | Lp | 0.003 | -92.6 | n.d | X | X | X |
| bOTU_2220 | c_Gammaproteobacteria | 0.815 | *P* = 0.009 | Lp | 0.008 | -57.44 | -94.46 | X | X | X |
| bOTU_2585 | o_Candidatus_Azambacteria | 0.815 | *P* = 0.018 | Lp | 0.006 | -95.55 | -86.49 | ✔ | X | X |
| bOTU_1899 | f_Gemmataceae | 0.813 | *P* = 0.020 | Lp | 0.004 | -12.33 | -68.93 | X | X | X |
| bOTU_664 | g_Methylobacterium | 0.812 | *P* = 0.041 | Lp | 0.014 | -49.79 | -38.29 | X | X | X |
| bOTU_8067 | c_Parcubacteria | 0.811 | *P* = 0.010 | Lp | 0.002 | n.d | -73.19 | X | X | X |
| bOTU_5229 | f_Gemmataceae | 0.801 | *P* = 0.025 | Lp | 0.002 | -73.54 | -72.55 | X | X | X |
|  |  |  |  |  |  | Lp | Tp |  |  |  |
| bOTU_12814 | f_Sandaracinaceae | 1 | *P* = 0.001 | Ci | 0.004 | -97.62 | n.d | ✔ | X | X |
| bOTU_3207 | g_Ilumatobacter | 1 | *P* = 0.001 | Ci | 0.005 | n.d | -96.8 | ✔ | X | X |
| bOTU_2679 | o_Planctomycetales | 0.971 | *P* = 0.001 | Ci | 0.008 | -88 | -77.27 | ✔ | ✔ | X |
| bOTU_7521 | g_Flavobacterium | 0.955 | *P* = 0.001 | Ci | 0.003 | -94.33 | -85.92 | X | X | X |
| bOTU_4645 | o_C0119 | 0.948 | *P* = 0.002 | Ci | 0.002 | -90.04 | -73.61 | X | X | X |
| bOTU_2992 | g_Nocardia | 0.942 | *P* < 0.001 | Ci | 0.003 | -94.85 | -79.84 | X | X | X |
| bOTU_8447 | g_Aeromicrobium | 0.938 | *P* < 0.001 | Ci | 0.013 | -89.76 | -71.1 | X | X | X |
| bOTU_6193 | d_Bacteria | 0.937 | *P* = 0.002 | Ci | 0.002 | -87.24 | n.d | X | X | X |

Table S5: continued

| OTU | Taxonomy | IndVal | *P* - value | Plant | ab. | % Change | | legacy | | |
| --- | --- | --- | --- | --- | --- | --- | --- | --- | --- | --- |
| Bacteria |  |  |  |  |  | Lp | Tp | June | August | September |
| bOTU_3778 | o_SBR1031 | 0.935 | *P* = 0.001 | Ci | 0.004 | -78.65 | -78.48 | X | X | X |
| bOTU_3788 | c_Parcubacteria | 0.932 | *P* = 0.001 | Ci | 0.006 | -87.12 | -86.54 | X | ✔ | X |
| bOTU_2619 | f_Burkholderiaceae | 0.930 | *P* = 0.001 | Ci | 0.006 | -83.26 | -76.22 | X | X | X |
| bOTU_3178 | d_Bacteria | 0.928 | *P* = 0.001 | Ci | 0.010 | -61.47 | n.d | X | ✔ | X |
| bOTU_1998 | c_S0134_terrestrial_group | 0.915 | *P* = 0.001 | Ci | 0.007 | -81.25 | -68.18 | X | ✔ | X |
| bOTU_958 | g_Georgfuchsia | 0.913 | *P* = 0.022 | Ci | 0.036 | -90.26 | -88.16 | ✔ | ✔ | X |
| bOTU_3010 | o_OPB41 | 0.909 | *P* = 0.002 | Ci | 0.006 | -87.99 | -82.11 | X | X | X |
| bOTU_2802 | g_OM60 | 0.908 | *P* = 0.001 | Ci | 0.012 | -89.91 | -74.79 | X | X | ✔ |
| bOTU_13291 | g_Pir4_lineage | 0.900 | *P* = 0.002 | Ci | 0.003 | -63.03 | -63.98 | X | X | X |
| bOTU_1647 | o_Candidatus_Yanofskybacteria | 0.900 | *P* = 0.008 | Ci | 0.016 | -70.21 | -94.6 | ✔ | ✔ | X |
| bOTU_12583 | f_Microscillaceae | 0.899 | *P* = 0.003 | Ci | 0.006 | n.d | -70.51 | X | X | X |
| bOTU_1603 | g_Lysobacter | 0.897 | *P* = 0.001 | Ci | 0.007 | -73.95 | -78.14 | X | X | X |
| bOTU_2908 | o_Planctomycetales | 0.892 | *P* = 0.004 | Ci | 0.005 | -83.29 | -69.63 | ✔ | X | X |
| bOTU_12471 | c_Parcubacteria | 0.890 | *P* = 0.001 | Ci | 0.001 | n.d | -58.29 | X | X | X |
| bOTU_9413 | d_Bacteria | 0.877 | *P* = 0.003 | Ci | 0.001 | n.d | -70 | X | X | X |
| bOTU_3515 | c_OM190 | 0.877 | *P* = 0.003 | Ci | 0.004 | -42.53 | -56.95 | X | X | X |
| bOTU_7951 | g_Herpetosiphon | 0.868 | *P* = 0.009 | Ci | 0.002 | -67.37 | -94.74 | X | X | X |
| bOTU_2502 | g_Parviterribacter | 0.866 | *P* = 0.005 | Ci | 0.006 | -77.66 | -75.09 | X | X | X |
| bOTU_3360 | f_BIrii41 | 0.866 | *P* = 0.005 | Ci | 0.003 | -94.57 | -71.56 | X | X | X |
| bOTU_3660 | o_SBR1031 | 0.866 | *P* = 0.006 | Ci | 0.003 | -95.92 | -77.9 | X | X | X |
| bOTU_5908 | f_Chitinophagaceae | 0.866 | *P* = 0.005 | Ci | 0.001 | -85.4 | -91.34 | X | X | X |
| bOTU_7019 | o_Babeliales | 0.866 | *P* = 0.006 | Ci | 0.001 | -57.6 | -83.33 | X | X | X |
| bOTU_7566 | g_Planctopirus | 0.866 | *P* = 0.005 | Ci | 0.001 | -82.24 | -49.69 | X | X | X |
| bOTU_8995 | o_Subgroup_7 | 0.866 | *P* = 0.005 | Ci | 0.002 | -87.75 | -80.21 | X | X | X |
| bOTU_8869 | g_Chthoniobacter | 0.865 | *P* = 0.006 | Ci | 0.003 | -37.92 | -46.44 | X | X | X |
| bOTU_1830 | o_Actinomarinales | 0.864 | *P* = 0.016 | Ci | 0.010 | -73.73 | -79.6 | X | X | X |
| bOTU_1446 | g_Gemmata | 0.864 | *P* = 0.016 | Ci | 0.011 | -68.21 | -83.48 | X | X | X |
| bOTU_2061 | f_Pedosphaeraceae | 0.857 | *P* = 0.011 | Ci | 0.009 | -84.22 | -88.83 | X | X | X |

Table S5: continued

| OTU | Taxonomy | IndVal | *P* - value | Plant | ab. | % Change | | legacy | | |
| --- | --- | --- | --- | --- | --- | --- | --- | --- | --- | --- |
| Bacteria |  |  |  |  |  | Lp | Tp | June | August | September |
| bOTU_1527 | f_Pedosphaeraceae | 0.855 | *P* = 0.006 | Ci | 0.007 | -72.07 | -68.87 | X | X | X |
| bOTU_7468 | c_Subgroup_6 | 0.855 | *P* = 0.040 | Ci | 0.010 | -90.42 | -61.7 | X | X | X |
| bOTU_3139 | d_Bacteria | 0.850 | *P* = 0.008 | Ci | 0.005 | -44.72 | -72.08 | X | X | X |
| bOTU_5122 | p_Patescibacteria | 0.850 | *P* = 0.009 | Ci | 0.003 | -47.84 | n.d | X | X | X |
| bOTU_1021 | g_Dinghuibacter | 0.846 | *P* = 0.049 | Ci | 0.014 | -71.69 | -54.07 | X | X | X |
| bOTU_9993 | g_Verrucomicrobium | 0.845 | *P* = 0.021 | Ci | 0.018 | -93.01 | -80.63 | X | X | ✔ |
| bOTU_3585 | o_Candidatus_Nomurabacteria | 0.845 | *P* = 0.003 | Ci | 0.003 | -89.99 | -95.62 | X | X | X |
| bOTU_3287 | o_Saccharimonadales | 0.844 | *P* = 0.044 | Ci | 0.011 | -91.72 | -73.31 | ✔ | X | X |
| bOTU_2511 | o_SJA-28 | 0.840 | *P* = 0.004 | Ci | 0.005 | -45.65 | -64.92 | X | ✔ | X |
| bOTU_3642 | f_KD3-10 | 0.837 | *P* = 0.016 | Ci | 0.005 | -80.78 | +30.03 | X | X | X |
| bOTU_10382 | f_Gemmataceae | 0.836 | *P* = 0.005 | Ci | 0.004 | -72.04 | -94.63 | X | X | X |
| bOTU_2818 | g_AKYG587 | 0.836 | *P* = 0.003 | Ci | 0.004 | -91.8 | -84.78 | X | X | X |
| bOTU_9217 | o_Candidatus_Kaiserbacteria | 0.835 | *P* = 0.010 | Ci | 0.002 | -58.32 | -84.36 | X | X | X |
| bOTU_3870 | o_Candidatus_Peribacteria | 0.831 | *P* = 0.003 | Ci | 0.001 | -93.06 | -41.73 | X | X | X |
| bOTU_4606 | g_Haliangium | 0.830 | *P* = 0.002 | Ci | 0.003 | -82.42 | -70.56 | X | X | X |
| bOTU_3669 | o_PeM15 | 0.830 | *P* = 0.016 | Ci | 0.003 | -56.84 | -90.58 | X | X | X |
| bOTU_5010 | f_Roseiflexaceae | 0.822 | *P* = 0.015 | Ci | 0.002 | -56.58 | -73.38 | X | X | X |
| bOTU_4449 | c_Parcubacteria | 0.821 | *P* = 0.006 | Ci | 0.001 | -85.89 | -61.67 | X | X | X |
| bOTU_4429 | f_Sandaracinaceae | 0.821 | *P* = 0.013 | Ci | 0.003 | -91.23 | -61.63 | X | X | X |
| bOTU_3324 | d_Bacteria | 0.820 | *P* = 0.006 | Ci | 0.003 | -74.9 | -91.8 | X | ✔ | X |
| bOTU_3860 | g_Starkeya | 0.819 | *P* = 0.016 | Ci | 0.006 | -83.47 | -73.4 | X | X | X |
| bOTU_2486 | p_WS2 | 0.817 | *P* = 0.004 | Ci | 0.004 | -43.54 | -63.22 | X | X | X |
| bOTU_5108 | c_Verrucomicrobiae | 0.816 | *P* = 0.015 | Ci | 0.002 | n.d | -79.76 | X | X | X |
| bOTU_1842 | g_Kamptonema_PCC-6407 | 0.812 | *P* = 0.013 | Ci | 0.010 | -86.31 | -88.47 | X | X | X |
| bOTU_2428 | o_Candidatus_Moranbacteria | 0.812 | *P* = 0.036 | Ci | 0.008 | -48.06 | -91.68 | X | X | X |
| bOTU_7459 | f_AKYH767 | 0.810 | *P* = 0.047 | Ci | 0.003 | +32.88 | +5.94 | X | X | X |
| bOTU_13242 | g_Nocardioides | 0.809 | *P* = 0.037 | Ci | 0.008 | -81.91 | -63.93 | X | X | X |
| bOTU_11428 | g_Ilumatobacter | 0.808 | *P* = 0.019 | Ci | 0.004 | -96.07 | -88.85 | ✔ | X | X |

Table S5: continued

| OTU | Taxonomy | IndVal | *P* - value | Plant | ab. | % Change | | legacy | | |
| --- | --- | --- | --- | --- | --- | --- | --- | --- | --- | --- |
| Bacteria |  |  |  |  |  | Lp | Tp | June | August | September |
| bOTU_4483 | f_WD2101_soil_group | 0.804 | *P* = 0.018 | Ci | 0.002 | -39.56 | -64.31 | X | X | X |
| bOTU_4730 | f_Gemmataceae | 0.803 | *P* = 0.015 | Ci | 0.002 | -77.14 | -95.19 | X | X | X |
| bOTU_1443 | g_Microcoleus_PCC-7113 | 0.801 | *P* = 0.008 | Ci | 0.016 | -65.86 | -88.71 | ✔ | X | X |
|  |  |  |  |  |  | Lp | Ci |  |  |  |
| bOTU_1479 | f_BIrii41 | 1 | *P* < 0.001 | Tp | 0.012 | -99.12 | -91.11 | ✔ | ✔ | ✔ |
| bOTU_3431 | g_Conexibacter | 0.969 | *P* < 0.001 | Tp | 0.007 | -83.76 | -74.33 | ✔ | X | X |
| bOTU_1468 | g_Bryobacter | 0.942 | *P* = 0.001 | Tp | 0.009 | -64.51 | -81.83 | X | X | X |
| bOTU_3082 | o_Solirubrobacterales | 0.942 | *P* = 0.001 | Tp | 0.009 | -85.93 | -80.41 | X | ✔ | X |
| bOTU_2392 | g_Jatrophihabitans | 0.926 | *P* = 0.001 | Tp | 0.011 | -76.22 | -81.58 | X | X | X |
| bOTU_5024 | g_SM1A02 | 0.926 | *P* = 0.001 | Tp | 0.001 | n.d | n.d | X | X | X |
| bOTU_1483 | g_Haliangium | 0.922 | *P* = 0.002 | Tp | 0.016 | -69.81 | -84.77 | X | ✔ | X |
| bOTU_2953 | o_Elsterales | 0.922 | *P* = 0.006 | Tp | 0.015 | -83.81 | -84.3 | X | ✔ | X |
| bOTU_2931 | f_Methylophilaceae | 0.919 | *P* = 0.003 | Tp | 0.007 | -92.2 | -92.06 | ✔ | X | X |
| bOTU_1121 | f_WD2101_soil_group | 0.918 | *P* = 0.001 | Tp | 0.013 | -62.6 | -95.48 | X | X | X |
| bOTU_790 | g_Acidothermus | 0.912 | *P* = 0.042 | Tp | 0.036 | -77.29 | -75.73 | X | X | X |
| bOTU_4803 | o_Candidatus_Nomurabacteria | 0.902 | *P* = 0.019 | Tp | 0.009 | -77.47 | -91.47 | X | X | X |
| bOTU_1518 | f_Micropepsaceae | 0.902 | *P* = 0.002 | Tp | 0.015 | -52.53 | -96.28 | X | X | X |
| bOTU_2282 | g_Haliangium | 0.900 | *P* = 0.005 | Tp | 0.003 | -83.71 | -86.04 | X | X | X |
| bOTU_1302 | f_Caldilineaceae | 0.896 | *P* = 0.003 | Tp | 0.009 | -63.77 | -88.72 | X | X | X |
| bOTU_7202 | f_TRA3-20 | 0.891 | *P* = 0.025 | Tp | 0.007 | -87.55 | -85.15 | X | X | ✔ |
| bOTU_1583 | f_Sandaracinaceae | 0.882 | *P* = 0.022 | Tp | 0.005 | -61.4 | -56.71 | X | X | X |
| bOTU_1094 | f_Acetobacteraceae | 0.879 | *P* = 0.009 | Tp | 0.016 | -60.12 | -95.08 | X | X | X |
| bOTU_2297 | f_P3OB-42 | 0.874 | *P* = 0.005 | Tp | 0.004 | -92.6 | -88.8 | X | X | ✔ |
| bOTU_1025 | g_Rhodanobacter | 0.873 | *P* = 0.013 | Tp | 0.017 | -86.35 | -93.7 | ✔ | ✔ | X |
| bOTU_2350 | g_Steroidobacter | 0.872 | *P* = 0.006 | Tp | 0.006 | n.d | -90.68 | ✔ | X | ✔ |
| bOTU_6934 | g_Luteolibacter | 0.863 | *P* = 0.009 | Tp | 0.004 | -89.57 | -82.86 | X | X | X |
| bOTU_4423 | f_AKIW781 | 0.852 | *P* = 0.015 | Tp | 0.014 | -64.29 | -60.38 | X | X | X |
| bOTU_3416 | f_Gemmataceae | 0.847 | *P* = 0.007 | Tp | 0.002 | -66.47 | -76.65 | X | X | X |

Table S5: continued

| OTU | Taxonomy | IndVal | *P* - value | Plant | ab. | % Change | | legacy | | |
| --- | --- | --- | --- | --- | --- | --- | --- | --- | --- | --- |
| Bacteria |  |  |  |  |  | Lp | Ci | June | August | September |
| bOTU_1426 | g_Rhodococcus | 0.845 | *P* = 0.007 | Tp | 0.006 | -84.35 | -84.49 | X | ✔ | X |
| bOTU_1498 | g_Rhodanobacter | 0.845 | *P* = 0.005 | Tp | 0.025 | -71.98 | -90.27 | X | X | X |
| bOTU_1683 | g_Ferruginibacter | 0.845 | *P* = 0.006 | Tp | 0.019 | -93.4 | n.d | ✔ | ✔ | ✔ |
| bOTU_1724 | g_Granulicella | 0.845 | *P* = 0.005 | Tp | 0.019 | -78.58 | -96.16 | X | X | X |
| bOTU_2022 | g_Candidatus_Solibacter | 0.845 | *P* = 0.006 | Tp | 0.010 | -65.12 | -95.85 | X | X | X |
| bOTU_2075 | o_Elsterales | 0.845 | *P* = 0.008 | Tp | 0.003 | -41.27 | -85.07 | X | X | X |
| bOTU_2878 | g_Gemmata | 0.845 | *P* = 0.005 | Tp | 0.003 | n.d | n.d | X | X | X |
| bOTU_4183 | o_Planctomycetales | 0.845 | *P* = 0.006 | Tp | 0.001 | -88.19 | n.d | X | X | X |
| bOTU_4258 | f_CPla-3_termite_group | 0.845 | *P* = 0.007 | Tp | 0.003 | -93.98 | n.d | X | ✔ | X |
| bOTU_7210 | g_Paenarthrobacter | 0.845 | *P* = 0.007 | Tp | 0.017 | -60.04 | -60.91 | X | X | X |
| bOTU_1746 | g_Gemmatimonas | 0.838 | *P* = 0.025 | Tp | 0.011 | -68.08 | n.d | X | X | X |
| bOTU_5898 | g_Burkholderia-Caballeronia-Paraburkholderia | 0.830 | *P* = 0.010 | Tp | 0.019 | -97.27 | -98.1 | X | ✔ | ✔ |
| bOTU_7172 | g_Pedosphaera | 0.825 | *P* = 0.017 | Tp | 0.007 | -74.87 | -90.31 | X | X | ✔ |
| bOTU_1798 | f_Xanthomonadaceae | 0.821 | *P* = 0.026 | Tp | 0.016 | -91.55 | -98.96 | ✔ | ✔ | ✔ |
| bOTU_2464 | o_Saccharimonadales | 0.810 | *P* = 0.013 | Tp | 0.003 | -68.43 | n.d | X | X | X |
| bOTU_1708 | f_Caulobacteraceae | 0.804 | *P* = 0.014 | Tp | 0.012 | -76.13 | -81.54 | X | ✔ | X |
| bOTU_2142 | f_Pedosphaeraceae | 0.801 | *P* = 0.031 | Tp | 0.008 | -68.99 | -95.48 | X | X | X |
